# Selection on Coding and Regulatory Variation Maintains Individuality in Major Urinary Protein Scent Marks in Wild Mice

**DOI:** 10.1371/journal.pgen.1005891

**Published:** 2016-03-03

**Authors:** Michael J. Sheehan, Victoria Lee, Russell Corbett-Detig, Ke Bi, Robert J. Beynon, Jane L. Hurst, Michael W. Nachman

**Affiliations:** 1 Neurobiology and Behavior, Cornell University, Ithaca, New York, United States of America; 2 Museum of Vertebrate Zoology, University of California, Berkeley, Berkeley, California, United States of America; 3 Ecology and Evolutionary Biology, University of Arizona, Tucson, Arizona, United States of America; 4 Centre for Proteome Research, Institute of Integrative Biology, University of Liverpool, Liverpool, United Kingdom; 5 Integrative Biology, University of California, Berkeley, Berkeley, California, United States of America; 6 Computational Genomics Resource Laboratory (CGRL), California Institute for Quantitative Biosciences (QB3), University of California, Berkeley, Berkeley, United States of America; 7 Mammalian Behaviour and Evolution Group, Institute of Integrative Biology, University of Liverpool, Leahurst Campus, Neston, United Kingdom; Stanford University School of Medicine, UNITED STATES

## Abstract

Recognition of individuals by scent is widespread across animal taxa. Though animals can often discriminate chemical blends based on many compounds, recent work shows that specific protein pheromones are necessary and sufficient for individual recognition via scent marks in mice. The genetic nature of individuality in scent marks (e.g. coding versus regulatory variation) and the evolutionary processes that maintain diversity are poorly understood. The individual signatures in scent marks of house mice are the protein products of a group of highly similar paralogs in the major urinary protein (*Mup*) gene family. Using the offspring of wild-caught mice, we examine individuality in the major urinary protein (MUP) scent marks at the DNA, RNA and protein levels. We show that individuality arises through a combination of variation at amino acid coding sites and differential transcription of central *Mup* genes across individuals, and we identify eSNPs in promoters. There is no evidence of post-transcriptional processes influencing phenotypic diversity as transcripts accurately predict the relative abundance of proteins in urine samples. The match between transcripts and urine samples taken six months earlier also emphasizes that the proportional relationships across central MUP isoforms in urine is stable. Balancing selection maintains coding variants at moderate frequencies, though pheromone diversity appears limited by interactions with vomeronasal receptors. We find that differential transcription of the central *Mup* paralogs within and between individuals significantly increases the individuality of pheromone blends. Balancing selection on gene regulation allows for increased individuality via combinatorial diversity in a limited number of pheromones.

## Author Summary

Individual recognition via scent is critical for many aspects of behavior including parental care, competition, cooperation and mate choice. While animal scents can differ in a huge number of dimensions, recent work has shown that only some specialized semiochemicals in scent marks are behaviorally relevant for individual recognition. How is individuality in specialized semiochemical blends produced and maintained in populations? At the extremes, individuality may depend on either a plethora of semiochemical isoforms or on combinatorial variation in a small number of shared isoforms across individuals. Analyzing the major urinary protein (MUP) pheromone blends of a wild population of house mice, we find evidence in favor of a combinatorial diversity model for the production and maintenance of individuality. Balancing selection maintains MUP proteins at moderate frequencies in the population, though interactions with the pheromone receptors appear to limit the extent of pheromone diversity in the system. By contrast, differential transcription of proteins greatly increases individuality in pheromone blends with balancing selection maintaining diversity in promoter regions associated with gene expression patterns. Selection maintaining combinatorial diversity in a limited set of behaviorally important semiochemicals may be a widespread mechanism generating and maintaining individuality in scent across taxa.

## Introduction

Animals produce complex blends of scents that can provide information on species, age, sex and individual identity [1]. As arguably the most complex and subtle form of recognition, individual recognition has attracted considerable attention from the perspective of neurobiology [2–4] and chemical ecology [5–7]. To be useful for individual recognition, scents must be variable among individuals in a population but stable within a particular individual over time [8–10]. Using discrimination tests, researchers have documented that a wide range of species are capable of differentiating between scents based on variation in multiple components of complex mixes of chemicals [8,11], leading to the early proposition that a wide range of genetic differences among individuals contributed to individual recognition [10,12–14]. Indeed many species produce consistent individual odor profiles that have the potential to mediate individual recognition [10,12–14], though the components of odors that are biologically relevant for individual recognition are generally unknown [15,16]. Recent studies using behaviorally salient tests of individual recognition rather than discrimination have identified specialized semiochemicals directly encoded in the genome as necessary and sufficient for individual recognition via scent marks in house mice [3,5,17]. These findings raise two important questions.

First, what is the nature of genetic variation underlying individually distinctive odor signatures? A key feature of individually distinctive scent profiles is their combinatorial nature, which could arise through a range of mechanisms including variation in coding sequences, gene transcription and protein translation. Determining the genotype-phenotype map has a number of important implications. The potential for pleiotropic effects varies between different elements of coding and non-coding DNA, which is expected to influence the rate of adaptive evolution [18]. Understanding the mechanisms giving rise to a phenotype are important for interpreting patterns of molecular evolution [19]. Finally, the relationships between chemical cues and genetic variation are widely discussed in the literature [12,20–22], but the mechanisms linking genotype and phenotype are generally unknown. Elucidating the nature of genetic variation underlying individually distinctive traits will provide insight into how well phenotypic differences could be used to infer genetic differences among individuals in a population.

Second, how is the diversity in specialized semiochemicals maintained? Models where individual recognition is mediated by a wide range of genes and metabolic processes propose that identity cue diversity is derived from neutral genetic diversity [23] or maintained by balancing selection on loci for other reasons, such as immune selection on MHC [24]. Thus, individually distinctive scent signatures have often been seen as identity cues rather than signals selected to advertise individual identity [14,24]. A separate line of research, however, argues that elevated diversity in phenotypes used for individual recognition is maintained because identity advertisement is favored when confusion among individuals results in costly misdirected behaviors, such as unnecessary aggression or missed mating opportunities [25–28]. Rare phenotypes are favored by negative frequency-dependent selection leading to the elevated diversity in identity signals [28,29]. Indeed, comparative phenotypic studies provide evidence that selection for identity signaling maintains variation in appearance [30], scent [31] or vocalizations [32,33] depending on the species. While selection for individual identity signals is expected to maintain increased diversity at relevant loci [34], molecular evidence of frequency-dependent selection on specialized semiochemicals mediating individual recognition in animals is lacking [24].

**Fig 1:**
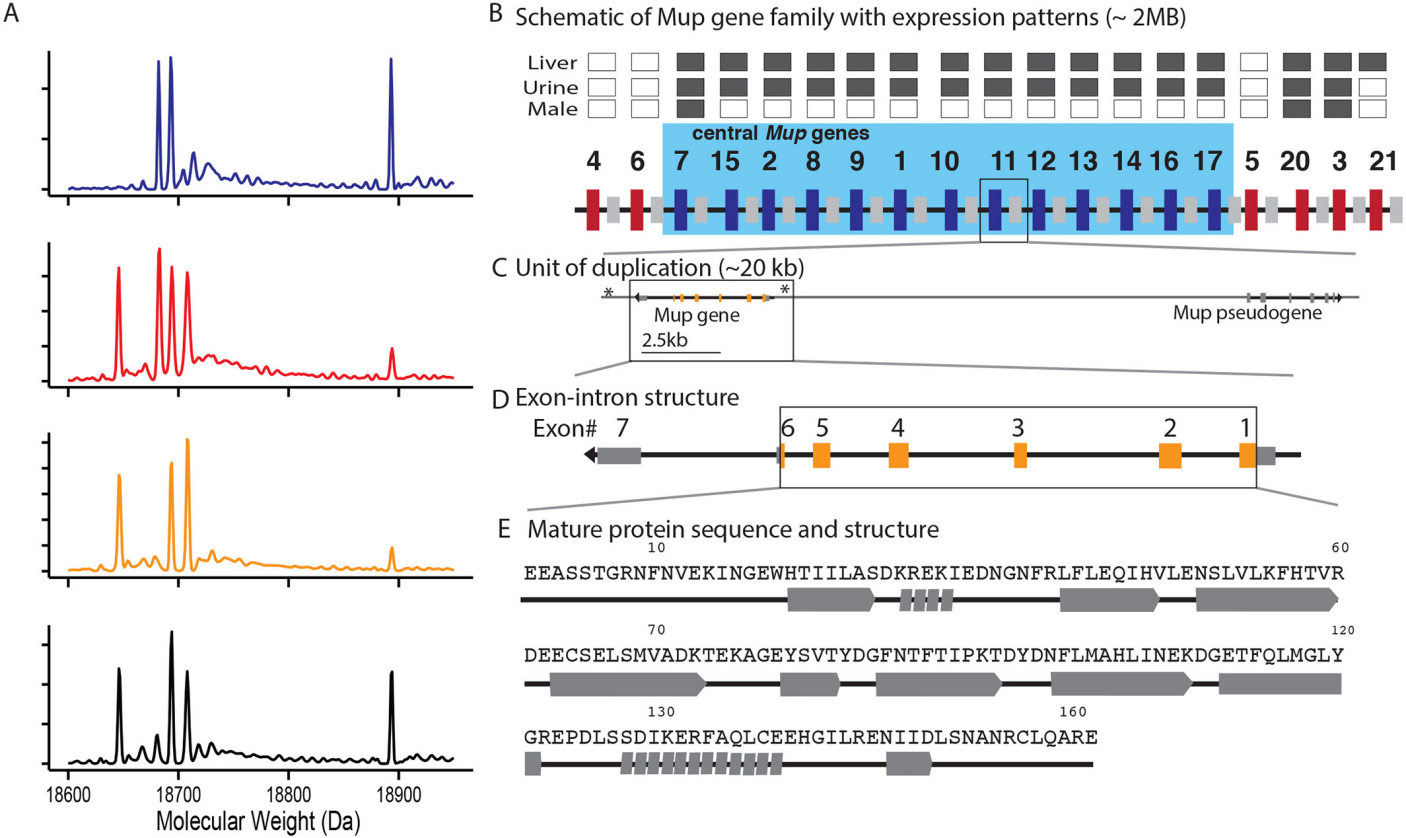
Major urinary protein phenotypes and gene family. Mice excrete individually distinctive combinations of major urinary protein isoforms in their urine, which are derived from the major urinary protein gene family. (A) Urine protein composition differs among individuals in both the presence/absence and relative of abundance of proteins in the urine. Proteins associated with individual identity have molecular weights from 18645 to 18724 Da and are the products of the central *Mup* genes. Each graph shows the mass spectrum for the urine of a different male from this study. The peak at 18893 Da corresponds to darcin, a specialized peripheral MUP (MUP20) that stimulates female memory of male scent. Panels B-E show the major urinary protein gene family structure at different scales. (B) The major urinary protein gene family consists of more than 20 members in the mouse reference genome. The panel shows a schematic representation of the layout of the gene family with central genes shown as blue bars and peripheral genes as red bars. Smaller gray bars denote pseudogenes. The chart above the diagram shows information on the expression of genes in liver and their excretion in urine in the reference mouse strain, C57BL/6, shaded in gray. We note which genes show male-biased expression in this strain. (C) The unit of gene duplication is ~20 kb and includes both a gene and a pseudogene. The location of primers used in this study is denoted by asterisks. (D) The intron/exon structure of *Mup11*, a representative central *Mup* gene. The portion of the gene that contributes to the mature protein is shaded orange. (E) The mature protein sequence of *Mup11* with the associated protein secondary structure. Alpha helices are denoted with a series of small parallelograms while beta sheets are denoted with large arrows.

In this study we examine the nature of genetic variation underlying individually distinctive urinary protein pheromones in a wild population of house mice and test for patterns of selection. The individual signature in mouse urine comes from combinatorial differences in the presence and relative abundance of isoforms of major urinary proteins (MUPs) (Fig 1A [3,5,35,36]). MUPs are detected by the vomeronasal organ and they also bind volatile pheromones that influence scent. The combinatorial protein variation mediates countermarking behavior in males as well as sexual preferences in females [3,37]. Previous work has demonstrated that MUP profiles are genetically determined and stable within adult individuals, and are highly heritable [5,17,38–40]. MUPs are the products of a family of tandemly-arrayed *Mup* genes and associated pseudogenes on chromosome 4, with more than 20 coding genes (Fig 1B and 1C [41–43]). The genes are broken into two categories based on their phylogenetic relatedness and sequence similarity, termed peripheral and central *Mups* corresponding to their position along the chromosome (Fig 1B). Variation in the excretion of proteins produced by the central *Mup* genes, which are >98% similar in coding sequence, is the main basis of individual variation in MUP phenotype in house mouse urine [3,41]. The nature of variation in central *Mup* genes among individuals in wild populations that gives rise to individuality of urine markings has yet to be determined.

Categorizing and analyzing molecular variation in dozens of nearly identical paralogs in natural populations presents a substantial challenge [44–46]. In the present work we sequenced large amplicons (~5kb) and developed approaches for analyzing patterns of variation among the highly similar central *Mup* gene complements across individuals. Though we are not able to assemble full gene sequences, our approach allows for tests of adaptive molecular evolution by comparing patterns of diversity among many different types of sites (e.g. non-synonymous, synonymous, etc.).

The present study seeks to determine the nature of genetic variation and assess what role, if any, selection on *Mup* genes has played in promoting and maintaining individuality in protein pheromone blends in wild house mouse urine. Using the F1 male offspring of unrelated wild-caught parents from one population (S1 Table), we dissect the contributions of variation in coding sequences, gene expression and protein translation to individuality in the urinary identity signals of house mice. We confirm a role for both differences in coding sequence and regulatory variation among the central *Mup* paralogs in generating individually distinctive MUP blends. Importantly, our work demonstrates that differential transcription of central *Mup* genes substantially increases differences in pheromone blends among individuals in a wild population. Analyses of sequence data provide evidence of frequency-dependent selection acting on both coding and 5’ regulatory sequences of the central *Mup* genes, with a particularly strong signal of selection on regulatory sequences. Taken together, these data highlight the importance of selection on gene expression in shaping combinatorial variation in pheromone blends used for individual recognition.

## Results

### Individual identity in MUP profiles of wild house mice

We measured the relative abundance of MUP isoforms at eight distinct molecular weights in the urine of 18 male laboratory-born descendants of wild mice (each individual from different wild-caught parents) using electrospray ionization mass spectrometry (ESI/MS). We collected and analyzed two urine samples from each sexually mature mouse (average age at time of urine collection = 218 days, range 190–251 days), which had all previously mated (S2 Table). The urine of mice in our sample shows the two hallmarks of an identity signal [5,47]. First, there is heterogeneity in the urinary protein profiles among mice; they differ in the presence as well as relative abundance of protein isoforms in mass spectra (Fig 1A). Second, each mouse produces a consistent pattern of urinary proteins (median Pearson correlation coefficient between relative abundance of protein masses in two independent samples from an individual: 0.98, range: 0.90–0.99). The urine samples analyzed from each individual were collected from 0–30 days apart, though there was no association between the time between urine samples and the similarity of those samples (S1A Fig, linear model, *t*_*16*_ = 1.45, *r*^*2*^ = 0.12, P = 0.17) demonstrating that the relative proportion of isoforms excreted in the urine is stable over time. Pairwise comparisons among the profiles of different mice show significantly lower correlations than between two different samples from the same mouse (S1B Fig, Kolmogorov-Smirnov test, D = 0.55, P< 0.0001, median Pearson correlation coefficient between samples from different individuals: 0.80, range: -0.08–0.99). Multiple MUPs with similar molecular weights (within the instrument capability of ±1–2 Da) are indistinguishable using the approach reported here and thus, variation between individuals is potentially underestimated using the ESI/MS methods of this study [48].

### Variation in coding DNA sequences contributes to individual identity

**Fig 2:**
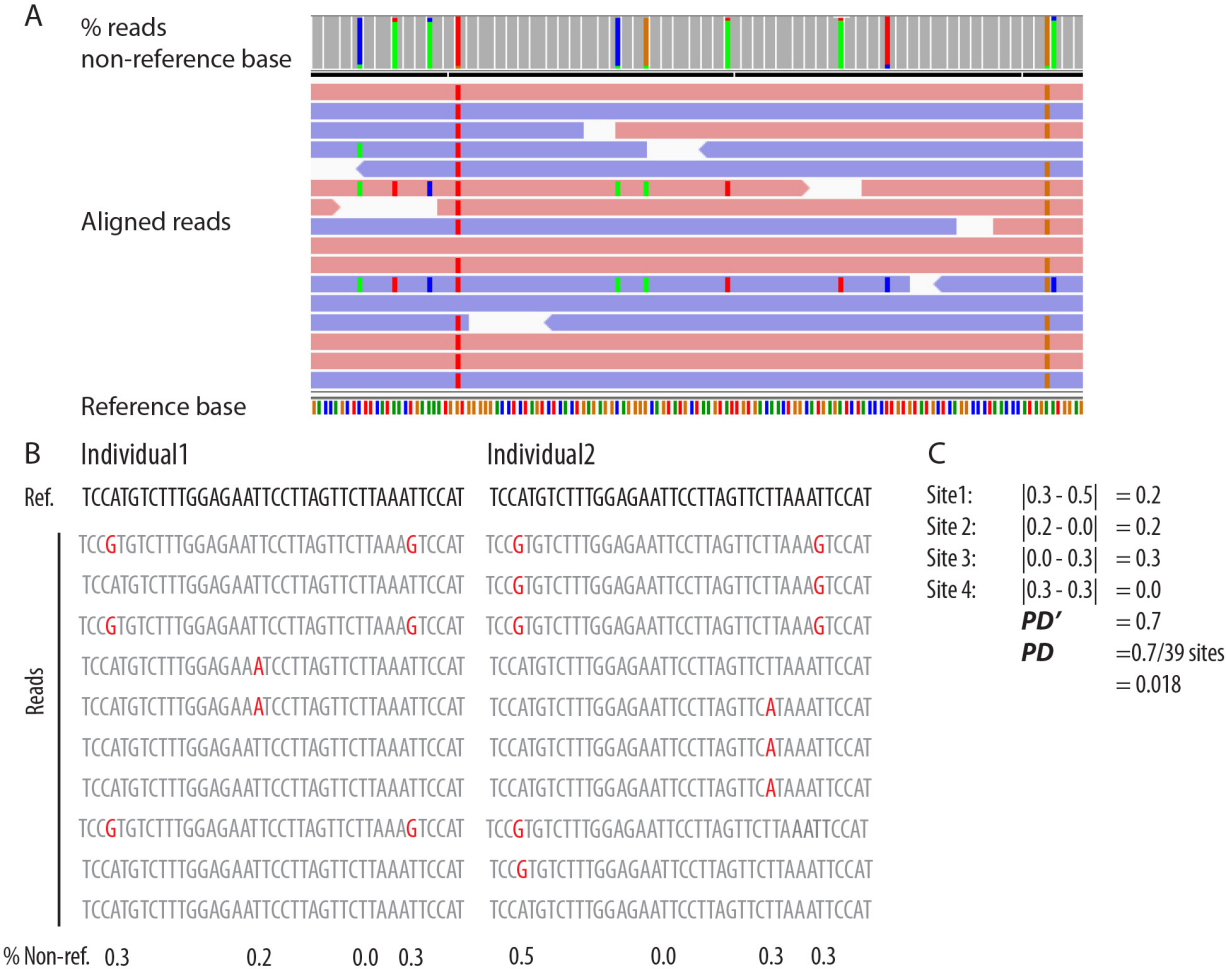
Strategy for aligning and analyzing variation to a single central *Mup*. (A) Cleaned reads were aligned to a single representative central *Mup* paralog, *Mup11*, resulting in average coverage >7000x across all individuals. The screenshot from IGV3.2 shows reads that come from multiple unique variants of the pictured region. The colored bars at the top show the relative proportion of non-reference reads across sites, which varies depending on the site. Data from the alignment of all central *Mup* reads to a single paralog was analyzed as the percentage of reads containing the non-reference base at a given site. Panel (B) presents a simplified hypothetical example of variation in sequences between two individuals, with variant sites highlighted red. (C) An example calculation of a pairwise difference index, *PD*, where the difference in the percent of non-reference reads is tallied across all variant sites between two individuals.

We used primers in conserved regions flanking the 5’ and 3’ ends of genes to amplify the central *Mup* genes, producing an amplicons of approximately 5Kb (Fig 1C). The high sequence similarity among the central *Mup* paralogs [41,42] prevented us from assembling full gene sequences or unambiguously aligning these reads to the reference genome. We took advantage of the high sequence similarity to develop a novel technique for analyzing sequence reads from the weakly diverged paralogs of multigene families (Fig 2A). Because the paralogs are so similar, reads from any one paralog readily align to all the others. Rather than aligning the reads to all of the central *Mup* genes found in the reference genome, we created a reference alignment file containing only one central *Mup* gene. Here we report data for the alignment of reads to *Mup*11 (nomenclature from the MGI database, [49]), although alignments to other central *Mup* genes produce the same results. The resulting alignment to a single central *Mup* gene provides information on the presence and relative abundance of mutations among all the paralogs amplified from an individual. This approach does not distinguish between allelic and paralogous variation, but it does provide a summary of all the *Mup* variation among mice that may contribute to individuality in MUP profiles. Hereafter, we simply refer to the combination of allelic and paralogous sequence differences as variants. The signal peptide at the N-terminal of the protein is cleaved prior to excretion in the urine [50,51], so we focus our results on the mature gene product that is excreted and can contribute to individuality in scent (Fig 1D and 1E).

Our data provide clear evidence for heterogeneity in coding sequences contributing to individuality of urine in wild house mouse populations. We find evidence for 11 segregating non-synonymous variants in the mature proteins, three of which are not found among the central *Mup* paralogs in the mouse reference genome (GRCm38[41])(Table 1). Three of the eleven variants are present in all the samples though occur at variable rates among individuals (Table 1). Variation in the percentage of non-reference reads may arise from the combination of a variable number of paralogs containing the mutation among individuals, allelic variation among paralogs, and variation in the total copy number of *Mup* paralogs among individuals. Our sequencing and alignment approach is not able to readily distinguish between these causes. The other 8 mutations are present in a portion of the individuals and tend to occur at low levels when they are present in fewer individuals (linear regression, *r*^*2*^ = 0.34, *t* = -2.15, *P* = 0.06), suggesting that rarer variants among individuals also tend to occur in few gene copies within individuals. All but one non-synonymous variant is shared by at least two individuals, though that variant occurs at reasonably high frequency in the one individual (19% of reads, Table 1). Collectively these data suggest that the 11 non-synonymous variants detected do not include any false positives. Furthermore, none of the variants detected are predicted to produce premature stop codons or frameshifts (Table 1), as would be expected if our alignment had contained sequences from *Mup* pseudogenes.

**Table 1:** Percentage of non-reference reads at non-synonymous variant sites in different individuals.

		Mouse ID^c^																	
Site^a^	Change	TAS285	TAS286	TAS287	TAS288	TAS289	TAS290	TAS291	TAS292	TAS293	TAS294	TAS295	TAS296	TAS298	TAS338	TAS339	TAS340	TAS359	TAS360
chr4:60662207	E 13Q	5				3				4								3	
chr4:60661804	D34E	28	1	1	22	29	1	9	1	18				1	15	1		18	8
chr4:60661803	N35H	28	1	1	21	29	1	9	1	18				1	15	1		18	8
chr4:60661769	H45R	5	12	9	12	3	11	3	9	6	8	7	7	8	4	8	6	4	3
chr4:60661756	N50K		7	10			11	14	7	4	10	13	9	11	4	10	13	5	14
chr4:60661740	F56V	28	34	20	21	27	19	28	21	23	16	14	17	17	21	16	13	21	28
chr4:60660909^b^	A77T	6				7				4					4			3	
chr4:60660903	E79K	4				4				2					3			3	
chr4:60660193^b^	D110N				19														
chr4:60659762	E140K	22	12	15	37	18	13	17	14	17	15	15	14	17	16	15	15	15	17
chr4:60659535^b^	R161L		5	10			8	7	9	3	8	7	7	9	4	8	7	3	7

a. All reads for the central *Mup* genes were aligned to the *Mup11* reference. Coordinates for the site from GRCm38.p3 mouse reference genome.

b. Variants not identified in previously published analyses of the mouse reference genome.

c. Columns each denote a unique mouse sample with the sample ID (e.g. TAS285, TAS286) that is used throughout.

All of the amino acid variants identified can be mapped to the structure of MUP 11 (1I04.PDB, [52]) (S2 Fig). With the exception of the frequently observed Phe->Val mutation at position 56 (mature protein numbering scheme), all of the amino acid substitutions are located on the surface of the protein, with one set of changes being located in a ‘patch’ at the position of two external loops. These changes are consistent with interactions with receptor molecules, and to some degree, with alterations in cavity volume and potentially, specificity of binding.

Using identical alignment and variant calling methods, we found evidence for 22 synonymous variants, of which nine were found only in a single sample with a relatively low percentage of reads (Table 2). Eight of these nine variants were from one mouse (individual TAS293) and were present at around 1%. These low frequency variants may be rare alleles or false positives. The particular mouse in question had higher than average read coverage (>10,000x) so it is possible that the ~1% non-reference reads are attributable to sequencing error which surpassed our cutoff (see Methods). Removing this individual (TAS293) from subsequent analyses of sequence variation related to selection did not affect the results. While it is possible that some of the synonymous mutations reported here are false positives, that synonymous mutations are at much lower frequencies than non-synonymous mutations is unambiguous in this dataset.

**Table 2:** Synonymous variants among the central *Mup* genes from different individuals.

	Mouse ID																	
Site^a^	TAS285	TAS286	TAS287	TAS288	TAS289	TAS290	TAS291	TAS292	TAS293	TAS294	TAS295	TAS296	TAS298	TAS338	TAS339	TAS340	TAS359	TAS360
chr4:660662227									1									
chr4:660662222									1									
chr4:660662221									1									
chr4:660662214									1									
chr4:660662208	3	5																
chr4:660661848		1	1		1	1			1				1					1
chr4:660661845									1									
chr4:660661831	18				20				11					12			11	
chr4:660661794			1			1			1									
chr4:660661792			1			1			1									
chr4:660661788									1									
chr4:660661782			1						1									
chr4:660661774			1						1									
chr4:660661748	1								1									
chr4:660661746									1									
chr4:660660943	24	47	38	12	20	45	12	36	30	39	40	40	38	27	37	40	27	13
chr4:660660209	11				13				7					8			7	
chr4:660660191		5	10			8				9	4	0	9		4	3		
chr4:660659782			1		1			1	1				1					1
chr4:660659774									1				1					1
chr4:660659750													1					
chr4:660659542									1									

a. All reads for the central *Mup* genes were aligned to the *Mup11* reference. Coordinates for the site from GRCm38.p3 mouse reference genome.

### Some *Mup* sequences are not expressed

Next, we sequenced liver transcriptomes, where urine-excreted MUPs are produced [53,54], and compared patterns of variants between DNA amplicons and the *Mup* transcripts to assess the role of gene expression in individual identity. To ensure deep sequencing of *Mup* transcripts, we size-selected cDNA libraries made from liver RNA to over-enrich *Mup* transcripts relative to the rest of the liver transcriptome. We followed the same approach used to align DNA, and we aligned RNA reads against all peripheral *Mup* genes with only *Mup11* representing the central genes.

**Fig 3:**
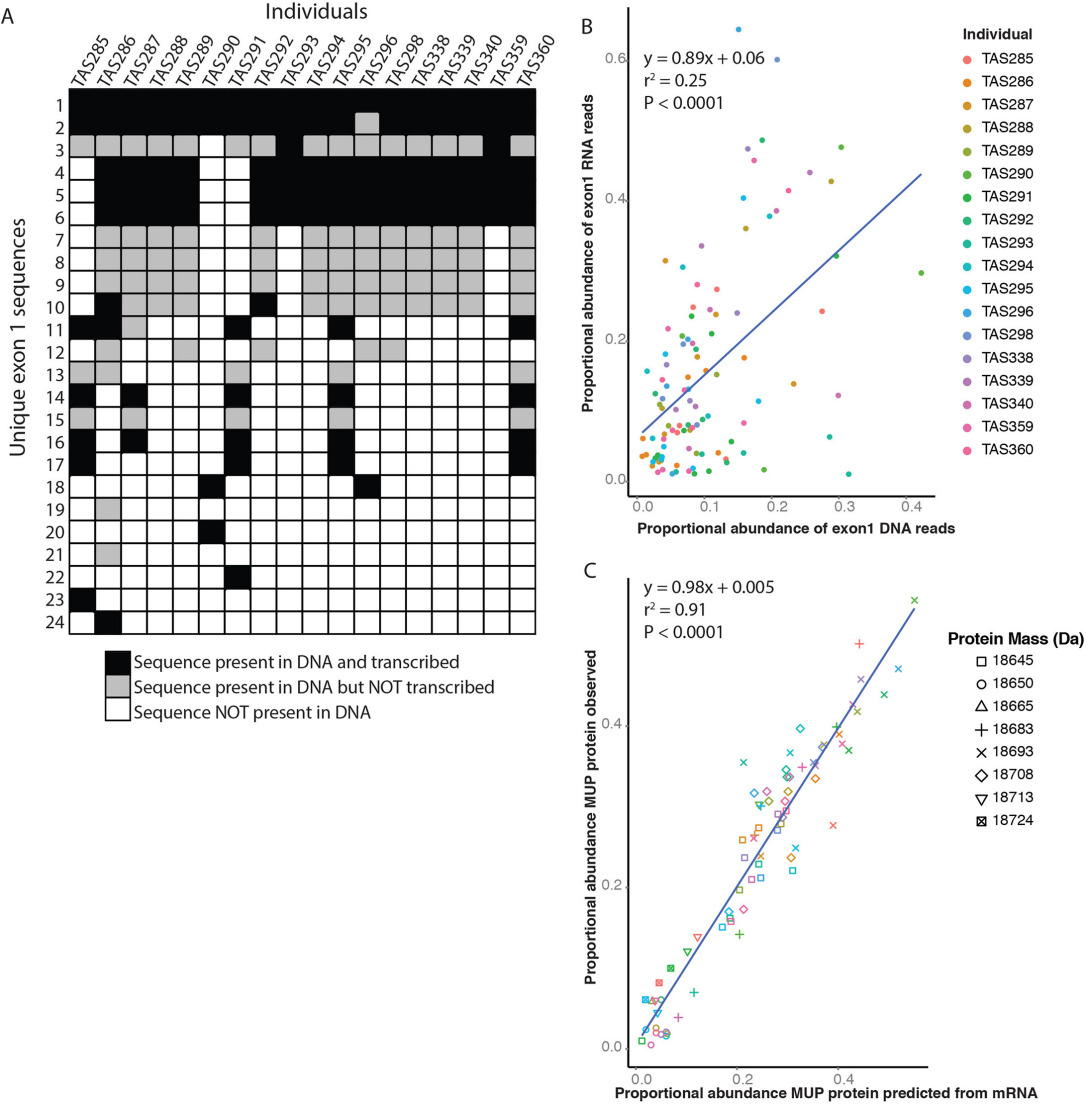
Genotype–phenotype map. Mice differ in the presence/absence and transcription of *Mup* genes. In panel (A) each individual is a column and each row represents a unique sequence of exon 1. (B) Among genes that are expressed within each individual, the relative abundance of DNA sequences is a modest predictor of the relative abundance of the same sequence in RNA in that individual. This pattern arises, at least in part, because for some genes present in multiple copies only a portion of the gene copies appear to be expressed. Points are colored according the individual mouse to which they belong. (C) The relative ratio of protein masses predicted by mRNA closely matches the observed ratios of masses in protein data, suggesting that post-transcriptional processes have little effect on individuality in mouse urinary scent marks. Points are colored according to the individual mouse to which they belong while the shape denotes the weight of the corresponding proteins. Note that some protein masses are generally more abundant in all individuals in the population than others.

Comparisons of RNA and DNA demonstrate differential rates of transcription among *Mup* sequences within and between mice. While it was possible to assemble long transcripts, our inability to generate full assemblies of DNA amplicons precludes direct comparisons of DNA and RNA across the entire length of the transcript. However, many reads span the full length of exons in both the RNA and DNA datasets; we compared abundances of unique reads of exon 1, the most variable exon, between the two datasets to assess patterns of transcription. Our data demonstrate that some of the DNA sequences identified in each mouse are not transcribed (Fig 3A). The lack of transcription raises the possibility that the sequences detected in the DNA data are actually pseudogenes, although two lines of evidence argue against this: (i) none of the variants in the DNA pool are early stop codons or frameshift mutations (Table 1) and (ii) the same sequences that are not transcribed in one individual sometimes show evidence of transcription in others (Fig 3A). Furthermore, previous studies of inbred lab strains have also demonstrated that mice do not transcribe all of their central *Mup* genes [41].

Overall, we find a weak positive relationship between the relative abundance of reads in DNA and RNA for exon 1 (linear regression, *r*^*2*^ = 0.26, *t*_207_ = 8.43, P < 0.0001). Even if DNA sequences that are not expressed (i.e., represent < 1% of RNA reads in a given individual) are excluded, the relationship remains modest. (Fig 3B, linear regression *r*^*2*^ = 0.25, *t*_101_ = 5.86, P < 0.0001). The positive relationship suggests that DNA sequences that occur in more copies tend to occupy a larger share of the mRNA pool.

### mRNA accurately predicts the pattern of excreted proteins

To assess the extent to which post-transcriptional processes such as variable translation or decay of proteins influence MUP phenotypes, we compared the predicted proportional abundance of MUP masses based on our mRNA data (S3 Fig) with the proportional abundance of urinary MUP masses measured by ESI/MS. The mRNA data very accurately predict the observed amount of each mass peak in a mouse’s urinary MUP profile (Fig 3C, linear regression, *r*^*2*^ = 0.91, *t*_73_ = 27.48, **B** = 0.98, *P* < 0.001), suggesting little if any role for variation in post-transcriptional processes in contributing to phenotypic differences among individuals. The tight relation between patterns of mRNA and urinary proteins, despite an extended period between collection of the urine and mRNA samples (average = 54.2 days, range 9–212 days), emphasizes the stability of the relative expression patterns of different MUP isoforms in adult mice and highlights their potential as an individual identity signal. Indeed, a statistical model explicitly incorporating time between urine and RNA collection shows that the tight relationship between the proportional abundances of mRNA and observed proteins does not change over time (P = 0.33). Furthermore, the ability of our method to analyze RNA to robustly predict the observed urinary protein patterns provides validation for the alignment methods employed in this study.

### Mutations in promoter sequences are associated with decreased expression

**Fig 4:**
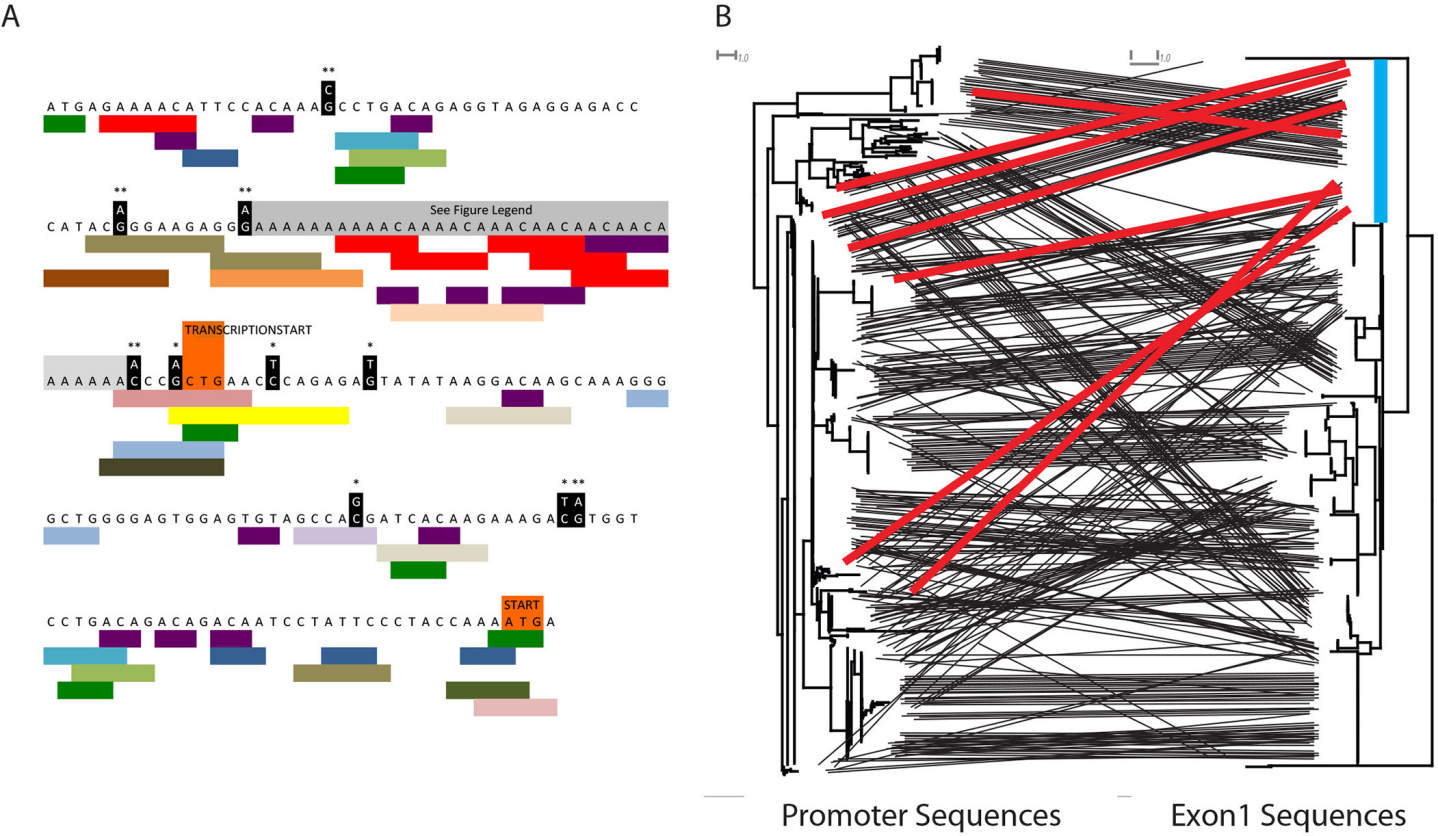
Variants immediately upstream of the start codon associated with expression. (A) Multiple variants immediately upstream of the start codon are significantly associated with reduced expression or silencing of genes. Variants significantly associated with reduced expression at P < 0.05 are highlighted in black (* < 0.05, ** < 0.01). Colored bars beneath the sequence are transcription factor binding site motifs, with each color denoting a different transcription factor. The adenine rich region shaded in grey has many substitution and indels found among promoter sequences in this study. Examples of the variants can be found in S3 Fig and have not been shown here because the poor alignment of sequences in this region complicates the associations of mutations at particular sites with expression data. (B) The tanglegram shows the relationship between promoter sequences and exon 1 sequences. Each tree is a neighbor-joining tree and identical sequences of a given exon or promoter are shown with independent lines either because they occurred in different individuals or because they were associated with different partners. The blue line in the exon tree highlights an abundant exon 1 sequence that is paired with divergent promoter sequences. The red lines show some of the connections between the blue-highlighted exon sequence and its promoters. The fact that a single exon sequence is associated with divergent promoter sequences is consistent with a history of recombination and gene conversion among the central *Mup* paralogs.

Differential silencing of some *Mup* paralogs could potentially be achieved through a diverse set of mechanisms, though variation in cis-regulatory regions seems likely because the relative expression of urinary protein isoforms is stable even as individuals alter their levels of total urinary protein output [5]. We examined a ~325 bp region including the first exon and upstream sequences for evidence of sequence variation associated with expression. Based on stringent criterion (see Methods) we classified promoters as expressed (N = 35) or silenced (N = 97). Using this restrictive dataset, we identified 10 eSNPs (Fisher’s exact test, P < 0.05) as being significantly associated with differences in expression category (Figs 4A and S3). We note that more variants are found only in the silenced category and also likely influence expression but are not common enough among the silenced promoters to register as significant given our restrictive dataset. Whether the mutations immediately upstream of the gene are causative is unclear, nevertheless they provide a potential mechanism for differential expression of *Mup* loci.

Despite silencing of some genes we do not detect frameshift mutations or early stop codons in the data set (Table 1), suggesting that although the upstream regions have become non-functional the exons have been maintained in a functional state. Comparisons of neighbor-joining trees for the promoter and exon 1 sequences demonstrate that a single exon 1 sequence may be associated with a diversity of promoter sequences across a population and within a single individual, consistent with a history of non-allelic homologous recombination (Figs 4B and S5).

### Differential expression of paralogs among individuals increases individuality

Variable gene expression could make individual scents more similar if shared sequences are expressed while rarer sequences are disproportionately silenced or under-expressed. Conversely, the preferential silencing or under-expression of common sequences would give disproportionate weight to rarer sequences resulting in greater phenotypic differences among individuals. To assess the extent to which variable gene expression either increases or decreases phenotypic differences among individuals, we compared the extent of differences between the same individuals at the DNA and RNA level. We calculated a pairwise differentiation index, *PD*, for all possible pairings of individuals by summing the differences in the percentage of non-references reads between individuals (Fig 2B and 2C). While *PD* ignores the linkage among variants (which we could not easily determine for DNA), it provides a reasonable proxy for the level of differences in urinary protein phenotypes based on DNA and RNA, especially when limited to non-synonymous variants that might lead to detectable scent differences among mice. While selection analyses should use values of *PD* corrected for the number of sites, we note that raw, uncorrected values of *PD* reflect total differences between two individuals and are appropriate for comparisons of predicted phenotypes based on DNA and RNA, especially given that the sequences compared are of the same length. To differentiate between the two uses of the index we define *PD*’ as the total difference between two individuals and *PD* as the difference between individuals corrected for the number of sites compared.

**Fig 5:**
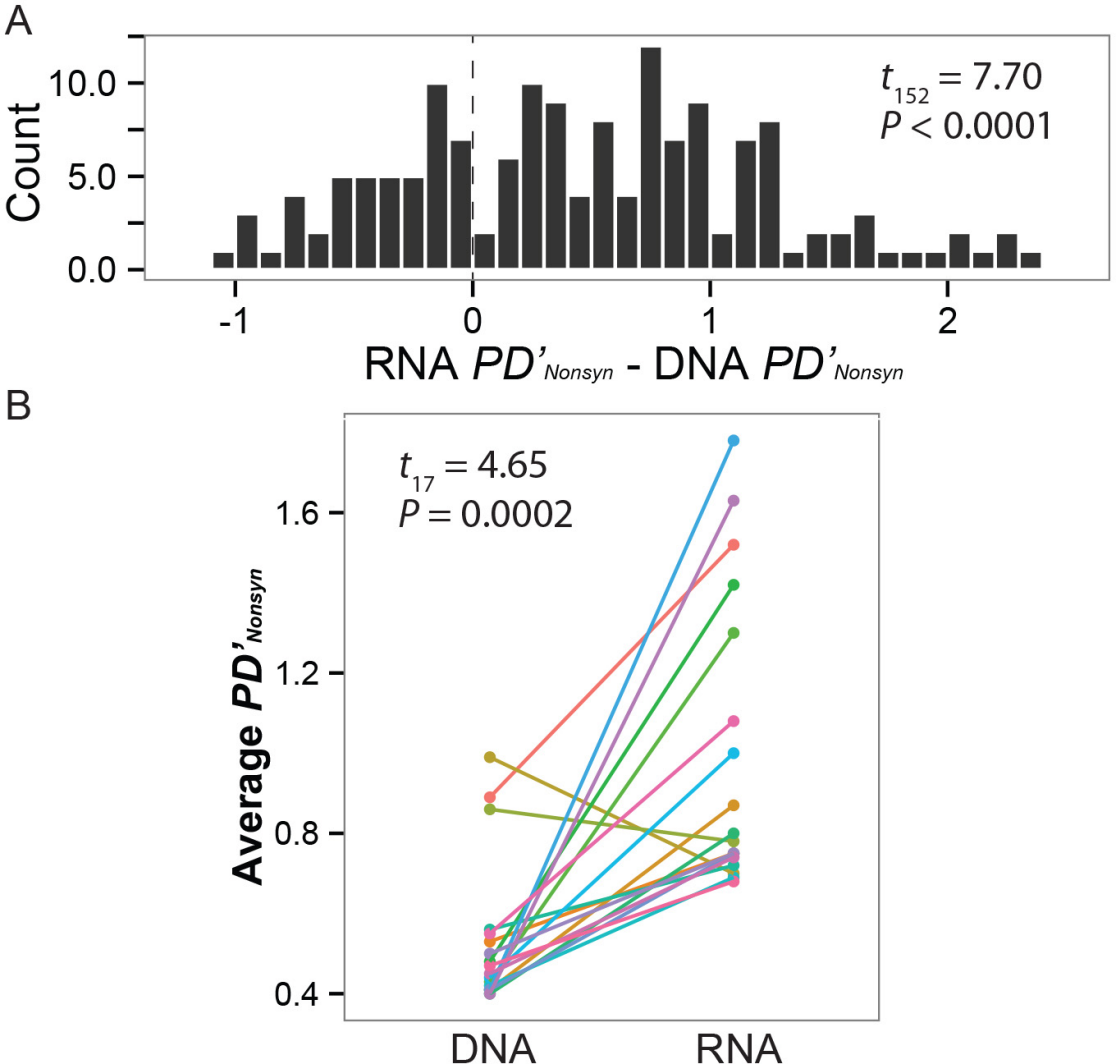
Variable expression of paralogs increases individuality. (A) The pairwise difference in non-synonymous sites, which would alter an individual’s protein complement, is higher in RNA than DNA. Values below 0 indicate that a pair of individuals is more similar at the level of RNA than DNA. Conversely, values above 0 indicate that individuals are more different as a result of differential transcription of *Mup* sequences. (B) A significant majority of mice experience an overall increase in individuality as a result of differential transcription.

Variable gene expression among individuals increases the differences between pairs of mice in a disproportionate number of pairwise comparisons (Fig 5A, Mann-mean RNA *PD’*–DNA *PD’* = +0.47, one-sample t test, *t*_152_ = 7.70, P < 0.0001). Though some pairs of mice end up more similar as a result of variable gene expression, in general mice are more distinctive at the RNA than DNA level. From the perspective of individual mice, we see an increase in average *PD’* for 16 of the 18 individuals as a result of variable expression of central *Mup* genes among individuals (Fig 5B, *t*_17_ = -4.65, P = 0.0002).

### Evidence for selection on coding sequences

Both coding and regulatory variation contribute to individuality, though it is unclear from our previous analyses whether the variation observed is consistent with neutral evolution of the *Mup* gene family or the result of selection for distinctiveness. To look for evidence of selection we compared patterns of variation at synonymous and non-synonymous sites. Though we do not know the precise number of gene copies measured across all individuals in our sample, it is constant across all the sites considered so variation in the percent of reads with a variant for each individual can be considered an estimate of the relative number of gene copies with a particular variant.

**Fig 6:**
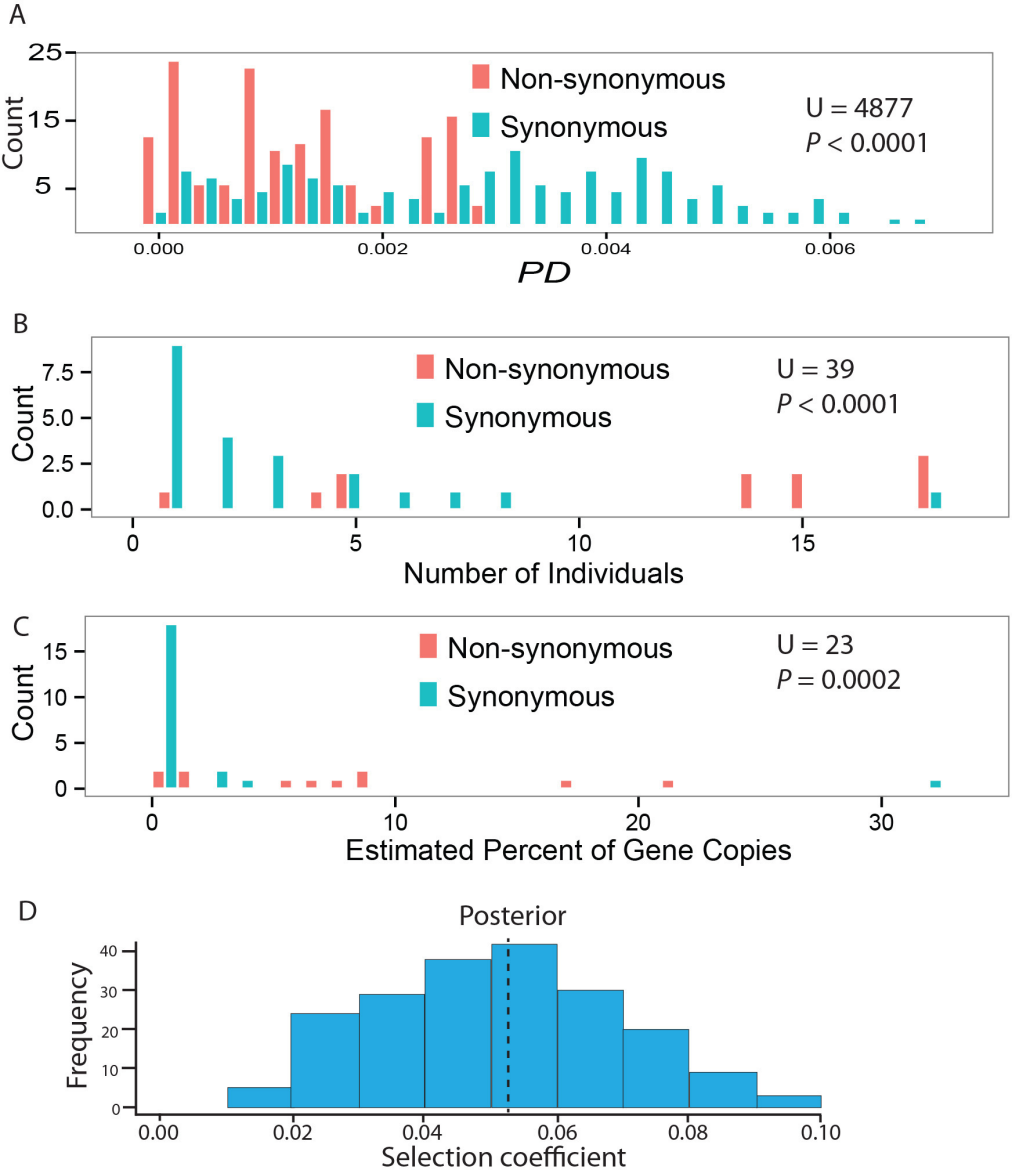
Selection on coding sequences. (A) Overall, there is higher diversity as measured by *PD* corrected for the number of sites in synonymous versus non-synonymous sites indicating purifying selection on the central *Mup* coding sequences as a whole. However, variation at a subset of non-synonymous sites appears to be under frequency-dependent selection. Consistent with frequency dependent selection, we find that (B) non-synonymous variants are found in more individuals and (C) occur at higher frequencies within individuals. (D) Simulations of the evolution of central *Mup* genes demonstrate that the patterns of variation observed here are inconsistent with neutral process and consistent with selection for distinctive phenotypes. The histogram shows the simulations that best fit the observed numbers and frequencies of non-synonymous and synonymous mutations. The fact that the best-fit simulations do not overlap zero argues for a role of selection in maintaining coding sequence diversity among the central *Mup* genes.

Overall, we find higher diversity in synonymous sites compared to non-synonymous sites (Fig 6A, median *PD*_*Non-syn*_ = 0.00127, median *PD*_*Syn*_ = 0.00328, MWU, U = 4877, n = 153 pairwise comparisons, P < 0.0001), consistent with high levels of purifying selection previously detected among the central *Mup* paralogs [55]. Though the overall coding sequence is under purifying selection, two observations suggest that non-synonymous variants may be under frequency-dependent selection. Non-synonymous variants are present in more individuals (Fig 6B, MWU, U = 39, P < 0.002) and occur at higher frequencies within individuals compared to synonymous mutations (Fig 6C, MWU, U = 23, P = 0.0002).

To investigate selection more formally, we conducted individual-based forward-time simulations of a single model of frequency-dependent selection using realistic demographic parameters. We then compared simulated numbers and frequencies of non-synonymous and synonymous mutations with observed values using approximate Bayesian computation [56]. The results of the simulations demonstrate that the patterns of variation described here are consistent with a history of relatively strong frequency-dependent selection maintaining diversity in central *Mup* genes at segregating replacement substitutions (Fig 6D). Importantly, the 95% credible interval for the strength of selection on MUP individuality signaling does not include zero (see Methods). Therefore, it is unlikely that neutral process could have generated the patterns of variation observed in the dataset. As with any simulation, the framework described here represents an approximation of the evolutionary processes that generated these patterns of genetic diversity in the central *Mups*. Nonetheless, these simulations provide support for the assertion that patterns of nucleotide variation are consistent with frequency-dependent selection maintaining non-synonymous diversity at select sites in an otherwise conserved gene sequence.

We also assessed the level of variation in the peripheral *Mup* genes, for which there is no current evidence of individual variability. Three of the genes sequenced, *Mup4*, *Mup5* and *Mup6*, are not expressed in the liver and not excreted in the urine [41]. Two of the peripheral genes, *Mup3* and darcin (*Mup20*), are expressed in the liver of males and excreted in the urine but serve separate signaling functions apart from individual identity. In males, both *Mup3* and *Mup20* elicit aggression [3]. *Mup20* mediates learned place preferences for the sites of previously encountered scent marks in both sexes [57] together with learned attraction to airborne scent from an individual male’s scent marks [58] and substantially enhanced neurogenesis in the hippocampus of females [59]. For three of the genes–*Mup3*, *Mup4* and *Mup5* –we did not identify any exonic variants in the population. We found two non-synonymous variants in *Mup6* and one in *Mup20*, all segregating at low frequencies (S3 Table). These observations provide no evidence for frequency-dependent selection on the peripheral *Mup* genes–indeed the lack of variation is indicative of purifying selection on the peripheral *Mup* genes. Instead, selection that maintains sequence variation appears limited to the central *Mup* genes involved in producing individually distinctive urinary scent marks.

### Evidence for selection shaping variable transcription of paralogs

**Fig 7:**
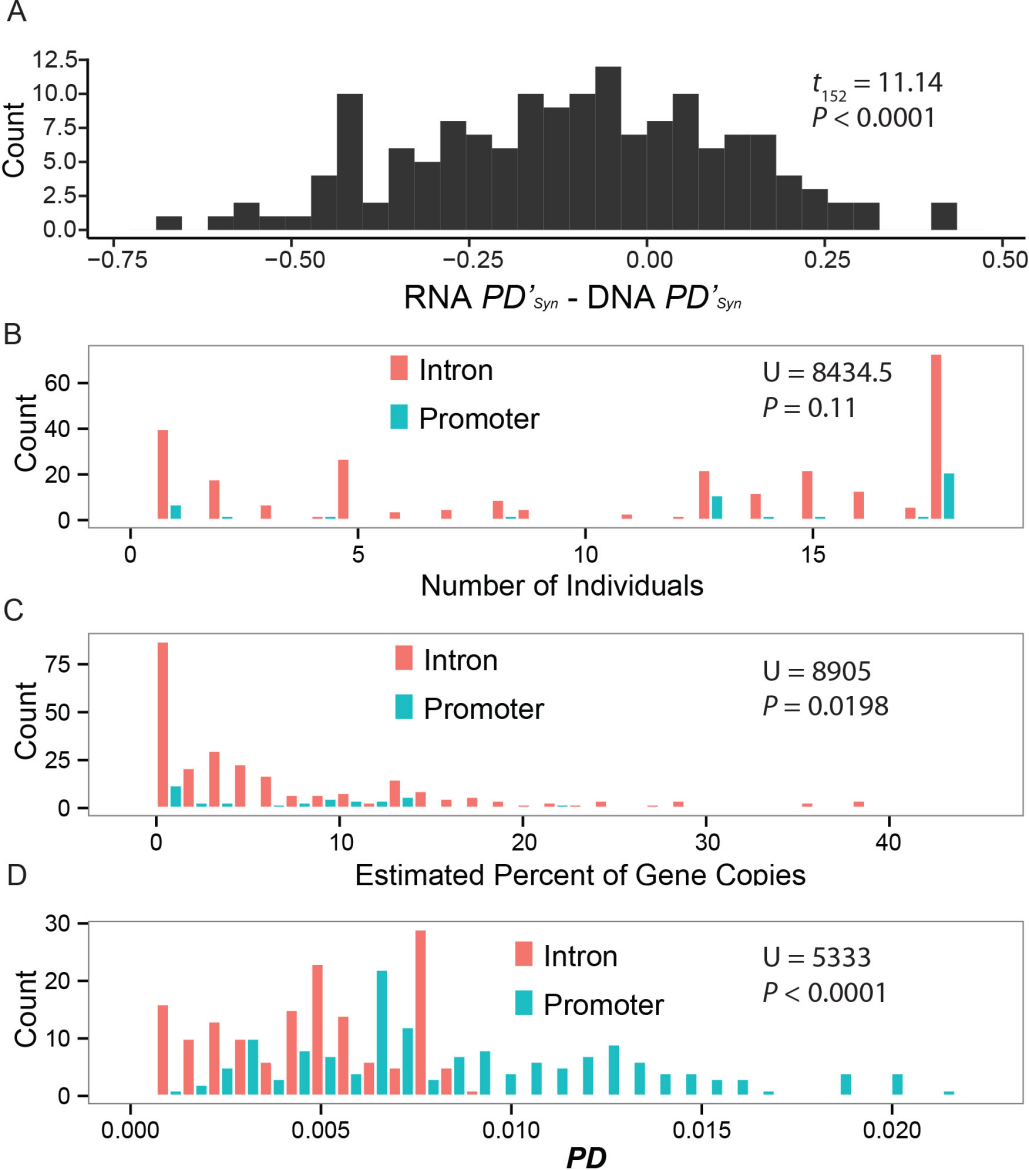
Selection on promoter sequences. (A) Variable gene transcription leads to a decrease in synonymous diversity in RNA compared to DNA, though the opposite pattern is found for non-synonymous sites. If variable gene expression were the result of a neutral process, it would be expected to influence patterns of diversity at non-synonymous and synonymous sites similarly. The increase in non-synonymous site individuality and simultaneous decrease in synonymous site individuality in RNA compared to DNA points to selection acting to increase individuality. Elevated sequence variation in promoter regions immediately upstream of the start codon provides additional evidence for frequency-dependent selection acting on regulatory sequences increasing individuality in urinary signals. (B) Compared to intronic variants, promoter sequence variants occur in similar numbers of individuals but (C) are found at more moderate frequencies. (D) Diversity measured as *PD* is much higher in the promoter region compared to introns.

While the increase in *PD’* between DNA and RNA is consistent with selection acting on regulatory sequences to increase individuality (Fig 5), it is possible that increased diversity merely results from the process of random mutations that silence genes or the duplication of already silenced genes. If this were the case, then we expect to see a similar increase between DNA and RNA in the synonymous differences among individuals, *PD’*_*Syn*_. Counter to the null prediction, we find a decrease in *PD’*_*Syn*_ in RNA compared to DNA (Fig 7A, MWU, U = 15783.5, P < 0.0001).

We also compared the diversity of promoter sequences to introns from the central genes. Variants in the ~200 bp region upstream of the start codon tend to be found in similar numbers of individuals as variants in the introns (Fig 7B, MWU, U = 8434.5, n = 55 promoter variants, 270 intronic variants, P = 0.11). However, promoter variants are found at higher frequencies compared to intronic variations (Fig 7C, MWU, U = 8905, P = 0.0198), consistent with selection maintaining diversity in promoter sequences. *PD* is greater in the promoter region compared to the introns (Fig 7D, MWU, U = 5333, n = 153 pairwise comparisons, P < 0.0001). This is consistent with diversity-enhancing selection on promoters or with lower constraint on promoters, although we note that introns are generally less constrained than 5’ flanking regions [60].

As with coding sequence diversity, comparisons between the central and peripheral *Mup* gene promoter sequences can determine whether the molecular patterns observed are specific to the central *Mup* genes involved in individual identity. Extensive promoter variation among the central *Mup* genes is unusual compared to the peripheral *Mup* genes, where we identified very little variation in promoter sequences (S3 Table). In both *Mup4* and *Mup6* we found no variants in the paralogous section of promoter sequence examined in the central *Mup* genes. We found one variant each in *Mup20* and *Mup5* segregating at low frequency (S3 Table).

## Discussion

### Genetic basis of identity information in MUP blends

Dissection of the genetic basis of individuality in the urinary scent marks of house mice demonstrates a role of selection on both coding and regulatory variation in maintaining adaptive identity-signaling phenotypic diversity. Our results confirm previously proposed roles for variation in gene sequences, the number of gene copies and transcriptional regulation in determining pheromone blends [41]. DNA sequences are weakly correlated with transcript abundance due to variable silencing of genes associated with mutations in promoter sequences (Figs 3 and 4). Whereas mRNA and protein abundances in general tend to be imperfectly correlated [61], we find a robust relationship between liver mRNA and proteins excreted in the urine across mice in our sample (Fig 3C). This is unsurprising, as MUPs are rapidly excreted from liver and there is no opportunity for intracellular degradation that can compromise the mRNA:protein ratio. This means that analysis of urinary MUP output is an accurate reflection of hepatic translational capacity, with the caveat that some distinct transcripts direct the synthesis of proteins of the same mature mass.

A number of studies have shown that the total amount of MUPs excreted in urine can vary over the course of an individual’s life [62–66] and between generations [67]. Thus, there is abundant evidence that the total amount of MUP excreted responds to social and physical conditions. However, the total amount of MUP does not provide identity information, in the same way that the volume of a voice does not identify a speaker. Thus, the critical question with regard to discrimination and recognition is whether the pattern comprising the presence and relative abundance of different MUP isoforms is consistent within individuals. The stability of MUP patterns for a given genotype is evidenced by the fact that individuals that share MUP haplotypes produce the same relative pattern (though not necessarily absolute amount) of MUP isoforms in their urine as adults [5,17,40,68,69]. A recent study using isoelectric focusing (IEF) to identify MUP patterns suggested that individual MUP profiles change over the course of adulthood [39]. However, the Thoß et al. study [ref 39] was based on unidentified gel bands that were not assigned to any gene products, confounding central MUPs, peripheral MUPs and any other urinary proteins of similar isoelectric point. Further, many of these bands were labeled as “minor” and it is likely that some were generated by artefactual changes caused by storage, notably deamidation of asparagine residues [70,71], an observation that caused us to abandon IEF as a method of profiling MUPs in all but freshly collected urine samples. Nonetheless, the Thoß et al. [ref 39] study still found a very high degree of consistency in bands expressed within individuals (median similarity of MUP profiles 94%, Figure 4 in ref [39]), confirming the long-term stability of the MUP pattern. Using genomic and ESI/MS methods, we provide clear evidence that proportional abundances of central MUP isoforms and corresponding liver *Mup* mRNA are very tightly correlated, even when measured more than six months apart (Fig 3C). Stable expression of identity signals is necessary for recognition, though identity signals need not be immutable to provide this function–for example, human faces have evolved to signal individual identity [34] and change to some extent with age, notably at sexual maturity. Electrophoretic studies show that expression of MUP isoforms develops progressively during adolescence [64,72], though they stabilize during adulthood [72]. Indeed, our transcriptomic data (and the tightness of the transcript:protein relationship) suggest that the proportional abundances of central MUP isoforms in adulthood are stable.

Differential transcription and silencing of central *Mup* genes among individuals is a major contributor to individual differences in patterns of urinary protein excretion. Previous discussions of chemical identity signatures have emphasized the importance of combinatorial information [3,24], though the genetic mechanisms that give rise to combinatorial variation had not been elucidated. By dissecting the relative contributions of variation in coding sequence, transcription and translation we have identified an important role of transcription in increasing individuality (Fig 5). We identify mutations in the promoter regions of *Mup* genes associated with a lack of transcription (Fig 4A), suggesting a mechanism through which regulatory changes lead to stable individual differences in patterns of urinary proteins. Chemical signatures of individual identity in other taxa, may similarly depend on regulatory variation to generate combinatorial semiochemical diversity.

### Scent signals of individual identity

The individuality in scent signatures has been widely viewed as a cue that arises through neutral process [23] or balancing selection on traits for reasons unrelated to recognition such as immune function [24]. Mice use the identity information in urine markings in a range of mouse social and sexual behaviors including territorial interactions and mate choice [17,73,74]. Given the importance of identity information to mouse social behavior, it is reasonable to hypothesize that MUP diversity has been selected to facilitate distinctiveness [28] and has evolved to signal individual identity. Theoretical models of identity signal evolution show that when confusion is costly phenotypes should be favored when rare as this makes them more distinctive [25,26,28], leading to frequency-dependent selection. The patterns of molecular variation presented here are consistent with a model where MUP diversity evolved to signal individual identity.

While the present data suggest that social and sexual interactions have favored the evolution of increased identity information in house mice, the relative importance of different behaviors that make use of identity information in selecting for diversity remains unresolved. In addition to their role in individual recognition [3,5,17], variation in central MUP isoforms has been shown to mediate assessment of heterozygosity [75], avoidance of inbreeding [76] and cooperation with relatives [40]. Therefore, it is possible that assessment of heterozygosity or the avoidance of inbreeding via disassortative mating [77] could be the mechanisms that maintain MUP diversity. In theory, a preference for novel MUP patterns could drive the evolution of extreme polymorphism as has been suggested to contribute to male color polymorphism in guppies [78,79]. Preferences for novel signals and disassortative mating, however, are unlikely to explain the diversity of MUP phenotypes in light of the facts that (i) unlike female guppies, female mice actually prefer familiar rather than novel males [74]; (ii) in wild populations, females also produce individually distinctive MUP patterns in their urine [40,72]; and (iii) the identity information in MUP patterns is used outside of the context of intersexual selection by males in territory marking and defense [3,5] and females in shaping patterns of cooperative nesting [40]. Alternatively, the maintenance of the MUP signals used to assess heterozygosity and recognize close relatives may be maintained because of selection for individual recognition via MUPs [28]. Models of genetic kin recognition argue against the evolution of genetic identity signals because cooperation among individuals with shared phenotypes is expected to erode signal diversity [80–82]. In contrast, models suggest that individual recognition is capable of maintaining signal diversity as common phenotypes are expected to suffer costs of confusion [25,26,28]. Of course, these alternative behavioral mechanisms favoring diversity are not necessarily mutually exclusive. Modeling studies and behavioral experiments are needed to partition the relative importance of different social or sexual contexts in maintaining MUP individuality.

### Evolutionary dynamics of the central *Mup* genes

Frequency-dependent selection may lead to balanced polymorphisms that maintain particular alleles for extended stretches of evolutionary time [83,84]. For example, correlated polymorphism in male coloration and mating tactics has been maintained for over 10 million years in multiple species of *Uta* lizards [85]. Alternatively, frequency-dependent selection coupled with high allele turnover when novel alleles are favored may result in accelerated divergence among alleles and lineages after speciation, as has been suggested for mating type recognition loci in fungi [86] and self-incompatibility alleles in plants [87]. The fact that central *Mup* gene sequences show signatures of purifying selection argues against a model of rapid allele turnover and diversification, though additional studies of *Mup* diversity in additional wild populations of *Mus* will be needed to clearly elucidate the evolutionary dynamics of the system. The dynamics may be complicated by selection on multiple gene sequences and regulatory elements as well as coding sequences.

A significant portion of the regulatory variation seen among central *Mup* paralogs is likely controlled by mutations in the immediate upstream region of the gene (Fig 4A). Despite many central *Mup* genes having apparently non-functional promoter sequences, they have not mutated into pseudogenes. This may be because they are expressed in other tissues at other times via a different set of regulatory sequences. They may also be maintained via ectopic gene conversion [88], which has been suggested to be an important force in the evolution of the central *Mup* paralogs [41,55]. In contrast, a previous analysis of the central *Mup* genes using GENECONV found little evidence for gene conversion [89], though as the authors of that analysis and others have pointed out, GENECONV can give misleading results when paralogous sequences are nearly identical, as is the case for the central *Mup* genes [89,90]. Comparing gene trees between subregions for incompatibilities, however, is a highly robust and powerful method for detecting gene conversion [90]. The fact that identical exon 1 sequences are coupled with divergent upstream sequences suggests a role for gene conversion reshuffling the arrangement of promoters and coding sequences in generating diversity. Thus, ectopic gene conversion may be a key mechanism for generating diverse expression profiles among *Mup* haplotypes.

Silenced gene sequences may act to enlarge the mutational target size for distinctive amino acid variants that are subsequently expressed as a result of gene conversion between expressed and silenced exons or the rescue of a silenced gene by conversion of the promoter sequence [91]. The evidence for selection maintaining variation in *cis*-regulatory elements does not exclude the possibility that *trans*-acting elements are also encoded within the *Mup* locus. Breeding experiments designed to explicitly test the interaction of haplotypes from natural populations will provide additional insight into the biology of the *Mup* locus.

### Implications for MUP-VNO interactions

The fact that the central *Mup* coding sequences as a whole are under purifying selection (Fig 6A) suggests that the overall protein sequence and structure are strongly conserved by selection. The restricted number of sites that show variation may arise due to constraints on the perception of urinary proteins. The individual identity signal in urinary protein blends is perceived by a subset of V2R receptors found in the vomeronasal organ with varying affinity to different isoforms [3]. Most of the non-synonymous variants in our sample are present among the paralogs in the mouse reference genome, though we did identify novel amino acid variants. Given the restricted geographic scope of our study, it is likely that broader sampling will reveal additional mutations as more wild mice are studied. Behavioral studies have suggested that mice are able to distinguish among many of the central MUPs encoded in the reference genome [3,5] and it is likely that the newly identified mutations produce perceptually distinctive proteins as well since the mutations are on the exterior of the protein (S2 Fig). Constraints on protein form may also arise because of selection on the ligand-binding pocket [55,89]. In addition to being directly detected by the VNO, MUPs also bind volatile molecules with isoforms showing differences in their specific binding affinities [92,93], which influence the volatiles held in scent marks [94].

Alterations to the coding sequence are only really beneficial if they bind to a distinctive set of V2R’s or influence volatile pheromone binding. The V2R’s belong to a large and dynamic gene family [95] but the subset related to detection of MUPs may similarly be under selection to be able to detect the diversity of pheromone blends in a population. Co-evolution between ligands and receptors has the potential to lead to rapid turnover [86,87], though the purifying selection observed on the *Mup* genes suggests that in this case receptor-ligand coevolution may lead to greater phenotypic stasis. Whereas changes to coding sequences have the potential to produce either a non-functional protein or one that is not perceptually distinctive, any substantial changes to the expression levels of genes will produce a new and distinctive combination of MUPs. Differential transcription greatly increases individual identity in the population examined here and may be especially important in the evolution of combinatorial recognition signals such as the MUPs.

### Population genetics of multigene families

As sequencing technology evolves, our ability to assemble full-length genes from complex multigene families from many individuals will improve, though we will still be faced with the challenge of interpreting patterns of variation. This problem is especially acute in tandemly-arrayed gene families such as the *Mup* locus where individual chromosomes differ in the number of gene copies present in the array [55,96], complicating patterns of paralogous and allelic variation. To address how the diversity of the central *Mup* genes shapes individually distinctive phenotypes we collapsed allelic and paralogous variation within the central *Mup* genes (Fig 2). From the perspective of mice assessing phenotypic variation in the blend of MUPs excreted in the urine, whether novel protein isoforms are generated by allelic versus paralogous differences is irrelevant. The *PD* statistic (Fig 2) provides a means for examining patterns of selection in such complex gene families. By comparing patterns of diversity in different parts of the genes, we provide evidence for frequency-dependent selection maintaining diversity in coding and regulatory sequences. The approach employed in this study holds promise for understanding the patterns of diversity in the *Mup* gene family in natural populations and may also be of use for other gene families where variation in the complements of proteins produced have fitness consequences, such as MHC.

### Conclusions

We provide molecular evidence for frequency-dependent selection maintaining MUP variation used for social recognition. As the variation among MUP isoforms is only known to function in scent communication [5,35,93,97], our results suggests that social interactions are sufficient to maintain genetic variation in recognition phenotypes. Analyses of molecular variation indicate that rates of divergence among urinary protein sequences are constrained, likely by their interaction with V2R receptors and the ability to bind low molecular weight ligands. The combinatorial ratios of isoforms are known to be behaviorally relevant [3], and accordingly our results highlight gene regulation as an important target of frequency-dependent selection in this system. Regulatory variation may be broadly important in maintaining individuality in combinatorial pheromone blends in other taxa as well.

## Methods

### Study subjects

We examined urinary protein variation in one F1 male descendant each from 18 unique crosses between wild house mice (*Mus musculus domesticus*) collected from the environs of Edmonton, Alberta, Canada (S1 Table). The wild caught mice were transported to the Animal Care facility at the University of Arizona, where the F1 male offspring were produced. The animal care and breeding protocol was approved by the University of Arizona Institutional Animal Care and Use Committee. We collected multiple urine samples from the same individual over the course of many days in the lab (see S2 Table for ages and sampling times). Upon being euthanized, mice were dissected, tissue was collected for subsequent extractions of DNA and RNA, and museum specimens were prepared. Specimens have been deposited at the Museum of Vertebrate Zoology at the University of California, Berkeley.

We focused on male mice as MUP concentrations tend to be higher in males compared to females and the roles of MUPs in male territory marking are well understood [3,5,67,98]. Urine and tissues were collected from mice after they were sexually mature and had been given the opportunity to mate. Urine samples and liver RNA were collected weeks apart (S2 Table). Previous research, however, has demonstrated that males maintain very stable MUP profiles in their urine throughout adulthood [5]. Indeed, our results indicate that the temporal separation of RNA and urine sample collection had minimal effect on the patterns of MUPs reported here.

### Urine collection and analysis

Urine was freshly collected from male mice by placing them in a clean cage; urine was pipetted once it was evacuated. Urine was briefly centrifuged to collect samples at the bottom of the tube and frozen at -80 C until subsequently shipped on dry ice to the University of Liverpool for analysis.

Total protein concentration in mouse urine was measured using a Coomassie Plus protein assay kit (Pierce, Rockford, USA), using bovine serum albumin as a standard. Urine samples were diluted in 0.1% formic acid to a concentration of 2 pmol/μl. All analyses were performed on a Synapt G1 Q-ToF mass spectrometer (Waters, Manchester, U.K.), fitted with an ESI source and coupled to a Waters nanoAcquity UPLC. Samples were injected onto a MassPrep C4 desalting column before elution over a 10 minute stepwise acetonitrile gradient, acquiring spectra between m/z 300–2000. Raw data was processed and transformed to a true mass scale using MaxENT1 maximum entropy software (Waters Micromass). All data sets were processed at 1 Da/channel over a mass range of 18,300–19,000 Da, and a peak width of 0.6 was used to construct the damage model. The peak lists of the true mass spectra were saved as text files and imported into SpecAlign [99] for spectral alignment. Baseline subtraction was by a factor of 20, an average spectrum was generated and spectra were aligned to this using the Combined (Correlation and Peak matching) Alignment method.

We tested for individuality by examining phenotypic correlations of urine protein composition within and between individuals. In the urine samples from our study population there were 8 well-supported ESI/MS peaks that correspond to the masses (in Da) of central *Mup* urinary proteins: 18645, 18650, 18665, 18683, 18693, 18708, 18713 and 18724. As our interest was in determining the consistency with which individuals reproduce the same blend of proteins, we used the ESI/MS peak heights associated with these protein masses to calculate the proportion of the total that was associated with each mass within a urine sample profile. We then calculated the correlation of the proportion of MUPs at each of the 8 masses between samples from the same and from different individuals and determined the Pearson correlation coefficients. Identity signals should be variable between individuals and similar within individuals so we tested for a difference in the correlation coefficient in the within *versus* between individual comparisons.

### Genomic DNA extraction, PCR and library construction

Genomic DNA was extracted from spleen using a Gentra Puregene Tissue kit (Qiagen). Traditionally, gene families have been studied by amplifying genes or exons using conserved primer sets followed by Sanger sequencing of clones [100,101]. This process is laborious and costly, especially for longer sequences requiring the design of internal primers and multiple rounds of Sanger sequencing. Additionally, cloning and Sanger sequencing has the potential to miss genetic variation without exhaustive sampling [100]. To reduce costs and to capture as much variation as possible, we devised a high-throughput sequencing approach for documenting variation among the *Mup* genes from multiple individuals derived from a wild population.

Four pairs of primers were designed for conserved regions upstream and downstream of all the *Mup* genes, generating a ~5kb fragment. Assembly and alignment of the reads to mouse reference genome (GRCm38) demonstrated that our PCR approach amplified most known members of the gene family (only *Mup*3 and *Mup*21, two divergent peripheral *Mup* genes, were not amplified) (Fig 1). Furthermore, the same primers successfully amplified PCR products in samples from distantly related mouse species, such as *Mus caroli*. Together these two pieces of evidence suggest that our approach is likely to have captured the overwhelming majority if not all of the diversity in the central *Mup* genes. To guard against any potential effects of PCR biases in the amplification of paralogs, we amplified each of the 3 primer pairs (S4 Table) in triplicate (for a total of 9 reactions) before combining the samples and constructing sequencing libraries. PCR conditions were as follows: (1) initial denaturation at 98 C for 2.5 minutes, (2) 25 cycles at 98 C for 10 seconds, 58 C for 30 seconds, 72 C for 3 minutes, (3) a final elongation at 72 C for 7 minutes. We also used 1 primer designed to amplify peripheral MUP genes and amplified it using 1 round of PCR following the same conditions. We used a high fidelity long range Taq for PCR (Platinum Taq, ThermoFisher).

To construct libraries we sheared the uncleaned PCR products using a BioRuptor, targeting fragments in the 600–1000 bp range. Libraries were constructed using the sheared amplicons following a custom protocol for generating individually-barcoded genomic libraries for Illumina sequencing [102]. Libraries were sequenced at the University of California, Davis Genome Sequencing Center using one lane of MiSeq (300pb paired end mode).

### Liver RNA extraction and sequencing

The MUPs excreted in urine are transcribed in the liver at high levels. At dissection, portions of the liver were stored in RNA-later kept at 4°C for one day and then frozen at -80°C until extracted. We extracted total RNA using the Qiagen RNeasy kit. As our goal was to sequence the liver mRNA with relatively long reads, we created double stranded cDNA libraries from RNA extracts rather than directly fragmenting RNA. Reverse transcription of full-length RNA has been shown to introduce biases into transcriptome libraries by failing to fully reverse transcribe long transcripts [103]. This is unlikely to influence our results because all the *Mup* transcripts are reasonably short and of similar length (~ 925 bp). While cDNA libraries can under-represent 3’ ends of long transcripts, all of the *Mup* transcripts are similar length and so should be similarly affected by any biases. As with the DNA amplicons, the cDNA samples were fragmented into 600–1000 bp range and then made into libraries using the Meyer-Kircher protocol [102]. Libraries were sequenced at the University of California, Davis Genome Sequencing Center using a MiSeq (300pb paired end mode).

### Alignment

Aligning either DNA or RNA reads to reference sequences presents a significant problem in the attempts to study population level diversity of multigene families. The high levels of similarity among the central MUPs (>98% coding sequence similarity) mean that reads from one central MUP paralog readily map to all of the paralogs. Furthermore, the high levels of sequence similarity and gene conversion among central MUP paralogs preclude the unambiguous assembly of reads into distinct paralogs. The same issues are not faced by the more divergent peripheral MUPs, which have lower sequence similarity and map uniquely.

To address alignment issues among the central *Mup* genes, we developed a novel approach for examining the DNA and RNA diversity among individuals in the population. Rather than aligning reads to the full complement of central *Mup* genes in the reference genome, we aligned reads to a single representative gene or transcript (*Mup11*) from the mouse reference genome (Fig 2). For each site we were able to compare the percent of reads that have the reference versus non-reference base across individuals. From these data we calculated a pairwise difference statistic, *PD*, described in more detail below and in Fig 2. We note that in the case of DNA, individuals vary in the number of copies of *Mup* genes [96] so that a given percentage of non-reference reads does not necessarily correspond to the same number of paralogs across individuals. For example, consider two individuals for which 20% of the reads are non-reference at a given site. If individual A has 25 central *Mup* paralogs across its two chromosomes and individual B has 20, the number of paralogs with non-reference bases would be 5 and 4 respectively. Raw reads were pre-processed with Cutadapt [104], FLASH [105] and Trimmomatic [106] to remove adapters, duplicate reads, contamination and to trim reads. Cleaned reads were aligned using default settings of Bowtie2 [107].

Commonly used variant calling programs assume a diploid state making them inappropriate for our alignments of multiple paralogs to a single reference paralog. We used the ‘count’ function in ANGSD to identify variants in our dataset [108]. The count function gives the number of reads reporting each base for a given site. The average read depth was >7000x for most individuals. Inspection of the distribution of read counts for each base showed very few sites with moderate read coverage for an individual (e.g. ~100x where typical coverage depth was >7000x for an individual). Therefore we used a hard cut-off and masked all bases with less than 100 reads. This approach could lead to false negatives or false positives though these should not be any more common at some sites compared to others and therefore are unlikely to substantially influence our results or conclusions (e.g. sequencing errors should occur evenly across non-synonymous and synonymous sites).

The peripheral *Mup* genes are single copy genes in the mouse reference and are assumed to be so in this analysis. The following peripheral *Mup* paralogs were amplified by our PCR reactions: *Mup4*, *Mup5*, *Mup6*, and *Mup20*. Two genes, *Mup3* and *Mup21*, were not amplified by our PCR reaction, though sequences from *Mup3* were available from liver transcriptomes. Therefore, we used standard variant calling methods in SAMtools [109] to generate a VCF file with the reads. We filtered putative SNPs based on quality, discarding any with a quality score of less than 100.

### Comparison of DNA and mRNA

To understand the relationship between DNA sequence and RNA abundance in our samples we compared the reads covering exon 1. We wrote a custom script to extract all of the reads that completely covered the exon (96–99 bp) in each dataset type and grouped identical reads using CD-HIT [110]. The script outputs two files providing the sequence and read depth of each unique sequence respectively. True variants are expected to have high coverage while erroneous sequences should have low coverage when the sequencing error rate is low and coverage is high [111]. Following [111], the list of putative exons for each individual was delineated by a sharp decline in read depth, with low coverage sequences assumed to be artifacts. We used the same procedure for determining putative exon 1 sequences in both the DNA and RNA datasets. All well-supported exon 1 sequences in RNA were independently identified as putative sequences in the DNA dataset, though some DNA sequences do not have corresponding sequences present in RNA (Fig 3A). In principle, the MUP variants observed in the DNA but not in the RNA (see Results and Fig 3) could be due to recombinant haplotypes artificially created during PCR, although several lines of evidence argue against this. First, previous studies have demonstrated that some MUP genes are not expressed [41], consistent with our findings. Second, some of the MUP variants that are not expressed in some individuals are nonetheless expressed in other individuals in our dataset (Fig 3A). Third, included among the variants that are uniquely found in the DNA are SNPs that are not found in the RNA; these cannot be a result of recombinant PCR. Finally we note, that the statistic *PD* does not take the phasing of haplotypes into account and so the potential for PCR recombinants would not affect the results of our population genetic analyses.

To correct for differences in sequencing depth among individuals and between the DNA and RNA datasets, we calculated the proportional representation of each well-supported exon 1 sequence, after first filtering out the reads designated as artifacts. We considered the relationship between the proportional representation of a given sequence in the DNA and RNA datasets for all the exon 1 sequences in DNA as well as the reduced subset that show evidence of expression (>1% of RNA reads within a given individual).

### Comparison of mRNA and protein

Of the ten amino acid substitutions found among the expressed central *Mup* sequences (S2 Fig), three cause only minor changes in molecular weight (~2 Da), preventing us from separating isoforms with and without these variants in ESI/MS spectra. Thus, we focused our comparison by collapsing the minor variation and considered the height of the following peaks: 18645, 18650, 18665, 18683, 18693, 18708, 18713 and 18724 Da (S3 Fig). This approach also allowed us to focus on a 131bp region of the transcript spanning exons 2 and 3. We assessed coverage depth of the 131bp region using a custom script that extracted all reads completely covering this region and grouped identical reads using CD-HIT [110]. From this output, we assigned the reads to an expected molecular weight and calculated the relative proportions of the weights. One amino acid variant outside of the 131bp region at the 3’ of the transcript (Table 1, S3 Fig, R161L) also has a measurable effect on the molecular weight of proteins. To account for this variant, we subtracted the read depth associated with this variant from the percentage of the relevant reads from the 131 bp section. We compared the predicted proportion of molecular weights to the observed ratios of the predicted proteins at the relevant peaks.

### Promoter regions

To examine the potential role of *cis*-regulatory mutations immediately upstream of the gene, we examined a ~325pb region including the first exon and approximately 200pb of upstream sequence (S4 and S5 Figs; note that the sequence length varies due to an A-rich tract of variable length just upstream of the gene). We matched previously defined putative exons with promoter regions when there was a perfect alignment between exon sequences. The >300bp region is longer than a single read and is only fully covered when the forward and reverse reads of the same sequence overlapped creating a longer contig. Thus, coverage depth for reads fully-spanning this longer region was low compared to the shorter stretch used to identify exons or predict molecular weights, so methods to identify sequences based on high coverage were not applicable [111]. Therefore, we considered a sequence to be a putative promoter sequence if the full 300pb region was present more than once in the dataset. This resulted in the identification of the promoter sequences for most exon 1 sequences.

We assigned promoter sequences as being expressed (>5%), weakly expressed (1–5%) or silenced (<1%) based on the proportional representation of the associated exon 1 in the RNA dataset. Many highly expressed exons are associated with multiple different promoters within a single individual, indicative of multiple gene copies. When expressed genes have a single promoter, one can unambiguously assign the promoter as being functional. Similarly, all promoter sequences associated with weakly or non-expressed sequences can be assigned to the lower expression category. By contrast, the presence of multiple promoter sequences makes it difficult to confidently ascribe function to any specific sequence. Therefore we did not consider groups of promoters associated with expressed genes. This left us with 20 promoters associated with exons with robust evidence of expression (>5%) and 112 promoters associated with weakly expressed or silenced exons (<5%). The high bar for being considered expressed is unlikely to wrongly assign any silenced promoters as being expressed, though could potentially mis-categorize some expressed promoters as being non-expressed. We aligned all the promoter sequences and found greater levels of non-consensus bases among the silenced promoters compared to the expressed promoters (S4 Fig). For each variant site, we used Fisher’s exact test to assess the association between the variant and expression phenotype. The position of variants relative to possible transcription factor binding sites in Fig 4 was determined using PROMO [112] with a cut off of 10% dissimilarity to the binding site motif allowed.

As noted above, single exon 1 sequences are associated with multiple different promoter sequences within the same individual. To assess the overall relationship between promoter and coding sequences, we constructed neighbor-joining trees for each part of the sequence in MEGA 5.2 [113] and created a tanglegram showing the links between the promoters and exons using Dendroscope [114].

### Testing for selection on the central *Mup* sequences

Since we were not able to align central *Mup* reads uniquely to reference genes, we could not use **π** or other widely used measures of sequence diversity to assess genetic variation within the sample. Instead, we calculated two metrics based on the logic of the site frequency spectrum and one based on **π**. We considered the distribution of variants in terms of the number of individuals in which the variant was detected as well as the overall percentage of reads in which it was detected. To calculate the overall percentage of reads, we summed the percentage of non-reference reads across individuals and divided by the number of individuals. Distributions of individual and percentage frequency spectra were compared among classes of sites using Wilcoxon tests.

Pairwise sequence differences among individuals can provide information on the abundance and frequency of mutations. Importantly, differentiation among individuals is critical to allow for identification based on scent. A difference in the percentage of reads with a particular variant between two individuals is reflective of differences in the relative number of gene copies or alleles containing that variant between two individuals. We summed the total differences in the percentage of non-reference reads across individuals and termed this the pairwise difference index, *PD*, as it measures the extent of differences between two individuals (Fig 2). As the value of *PD* is determined for each pair of individuals, we chose to represent it by its distribution across all pairwise comparisons in our dataset (N = 153) and compared differences in the distributions using Wilcoxon tests. We use the notation *PD’* to denote values that compare the total difference between two individuals uncorrected for the number of sites examined and *PD* to denote site-corrected comparisons.

### Simulations

We used individual-based forward time simulations to provide additional tests of selection for our dataset. Simulations were written in C++ and the source code is available in S1 File. Briefly, we simulate a Wright-Fisher population of diploid individuals wherein each chromosome has a cassette of 15 MUP proteins as in the reference genome. All rate parameters are based on expected values for *Mus* scaled by 100. We then allow gene conversion between any two MUP paralogs at a rate of 1e-4 with a conversion tract length drawn from a geometric distribution of mean 50. Recombination occurs between homologous MUP proteins at a rate of 5e-6/cassette/individual/ generation. These conditions fix the cassette size at 15. Although the cassette length undoubtedly varies in natural populations [96], and this may be an important source of functional variation for diversifying selection, there are few models of cassette size mutation precluding us from analyzing this aspect of functional evolution of MUP proteins.

We then allowed mutations at 15 total non-synonymous sites and 102 synonymous substitutions at a rate of 3e-6/site/generation. All other non-synonymous sites are assumed to be deleterious and are therefore not expected to experience diversifying selection, consistent with sequence similarity of MUP proteins. Negative frequency dependent selection is applied as follows: every generation the mean frequency per individual of each non-synonymous mutation is computed. We then compute the total difference in frequency between each individual’s genotype and the mean genotype across all non-synonymous sites. The fitness of the maximally different individual is equal to one, and an individual of exactly average allele frequencies is fitness 1 –s. The fitness of all other individuals is based on their total allele frequency differences relative to the average frequencies and distributed linearly between 1 and 1—s.

We conducted 10,000 simulations wherein we drew s from a uniform distribution between 0 and 0.1, and the effective population size from a uniform distribution between 100 and 1,100. These prior bounds were selected after 1,000 simulations with much wider priors suggested the maximum *a posteriori* estimate would be within each interval. Each simulation was run for 10N generations. We then used ABCreg [115] to regress the following four summary statistics onto the data: the number of non-synonymous and synonymous segregating sites in a sample of 18 individuals, and the mean frequency of synonymous and non-synonymous substitutions. We used the tangent transformation in ABCreg, and defined the tolerance at 0.02 to obtain the posterior distribution of selective coefficients. We estimated the maximum *a posteriori* estimate using the locfit package in R [116].

In an effort to be conservative in our inference of selection operating on non-synonymous polymorphisms in *Mus*, we also performed an additional 10,000 neutral simulations. We then combined these neutral simulations with the 10,000 simulations described above to produce a set of results based on a highly neutrality-biased prior distribution. Based on this very conservative prior distribution, and using the same regression approach as above, the resulting credible interval still does not include s = 0.

## Supporting Information

S1 Fig Individuality in mouse urine. (A) The time between sampling does not influence the relative pattern of MUP isoforms in a given mouse’s urine. The high correlation coefficients approaching one indicate that two samples taken days apart from the same mouse are very similar. (B) Urine samples from the same individual (Self) are more similar in their protein profile compared to two samples collected from different individuals (Different Mouse). The broad distribution of values for correlations between different mice shows that while some mice have very dissimilar urinary profiles, others are somewhat similar. Overall, however, different mice produce distinctive urinary scent marks.
(TIF)

S2 Fig Position of amino acid variants in the mature protein. Ten of the eleven amino acid variants occur on the outside of the protein consistent with a role for the variants in differential binding between MUPs and type 2 vomeronasal receptors (V2Rs).
(TIF)

S3 Fig Central *Mup* transcripts. The mice in our sample collectively excrete 17 unique proteins in their urine, many of which are not found in the reference genome. The signal peptide of exon 1, the sequence of which is identical between some genes, is not shown here. The dashed line indicates the region used to estimate the expected molecular weight proportions from RNA data. The * denotes a site which is variable in the DNA data but is not variable in the RNA dataset.
(TIF)

S4 Fig Central *Mup* promoter sequences. An illustrative sample of promoter variants found just upstream of the start codon in our sample. Variable sites are highlighted in grey. The expressed sequences (top group) show little variation compared to the silenced sequences (bottom group). The large, variable poly-A region lies immediately upstream of the transcription initiation site.
(TIF)

S5 Fig Expressed and silenced promoters associated with the same exon 1 sequence in one individual. Promoter sequences associated with the same exon 1 sequences (large white block) from a single individual suggest that some copies of multi-copy genes are silenced while others are expressed. The exact expression state of the promoter shown here is not known as it is not possible to differentiate among the copies in the RNA pool. The expression state is inferred based on the similarity of the promoter sequences to sequences of known expression status.
(TIF)

S1 Table Samples used in this study. (DOCX)

S2 Table Timing of urine and RNA sampling. (DOCX)

S3 Table Peripheral *Mup* variants. (DOCX)

S4 Table Primers used in this study. (DOCX)

S1 File Code used for computer simulations. (CP)

S1 Dataset (CSV)
