# Supplementary figures and images for: Selection on Coding and Regulatory Variation Maintains Individuality in Major Urinary Protein Scent Marks in Wild Mice
Source: PLoS Genet. 2016 Mar 3;12(3):e1005891. doi: 10.1371/journal.pgen.1005891 (PMC4777540; doi:10.1371/journal.pgen.1005891)

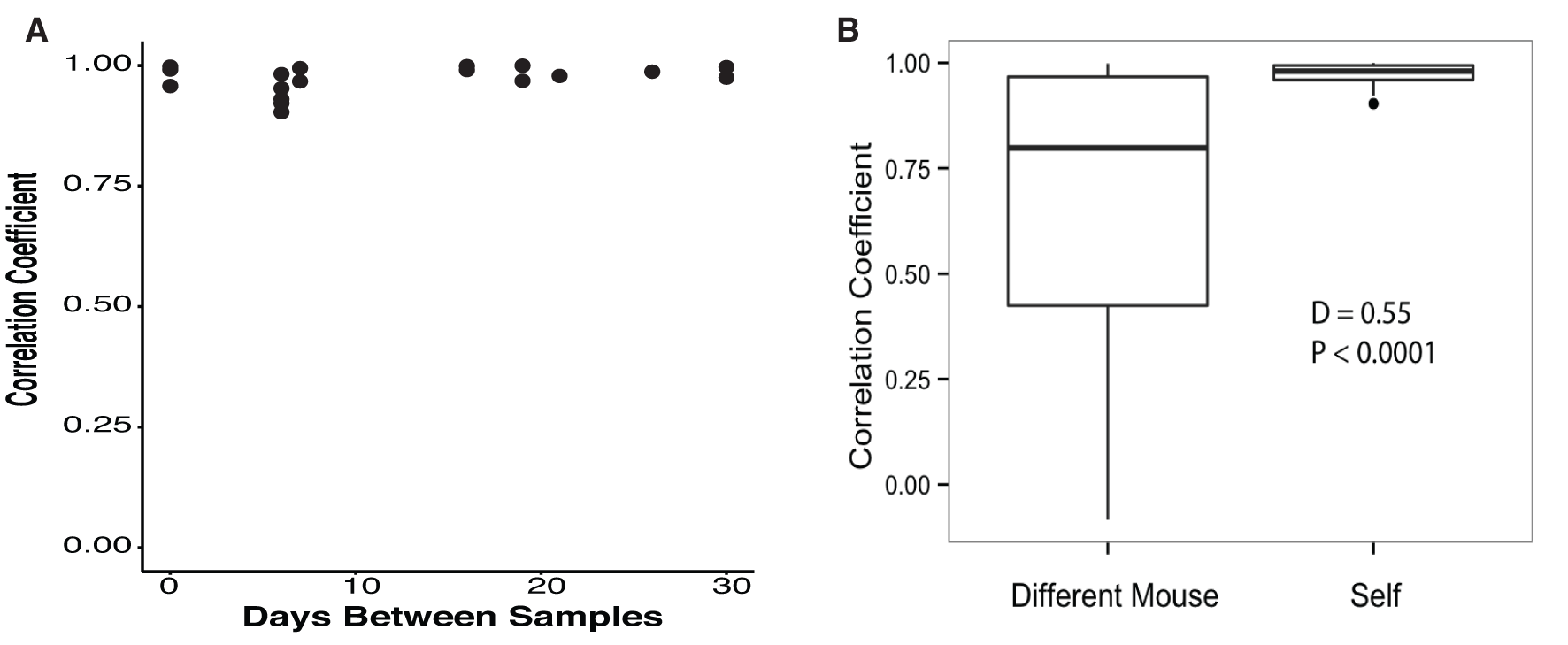

Supplement: S1 Fig — (A) The time between sampling does not influence the relative pattern of MUP isoforms in a given mouse’s urine. The high correlation coefficients approaching one indicate that two samples taken days apart from the same mouse are very similar. (B) Urine samples from the same individual (Self) are more similar in their protein profile compared to two samples collected from different individuals (Different Mouse). The broad distribution of values for correlations between different mice shows that while some mice have very dissimilar urinary profiles, others are somewhat similar. Overall, however, different mice produce distinctive urinary scent marks. (TIF) [file pgen.1005891.s001.tif]

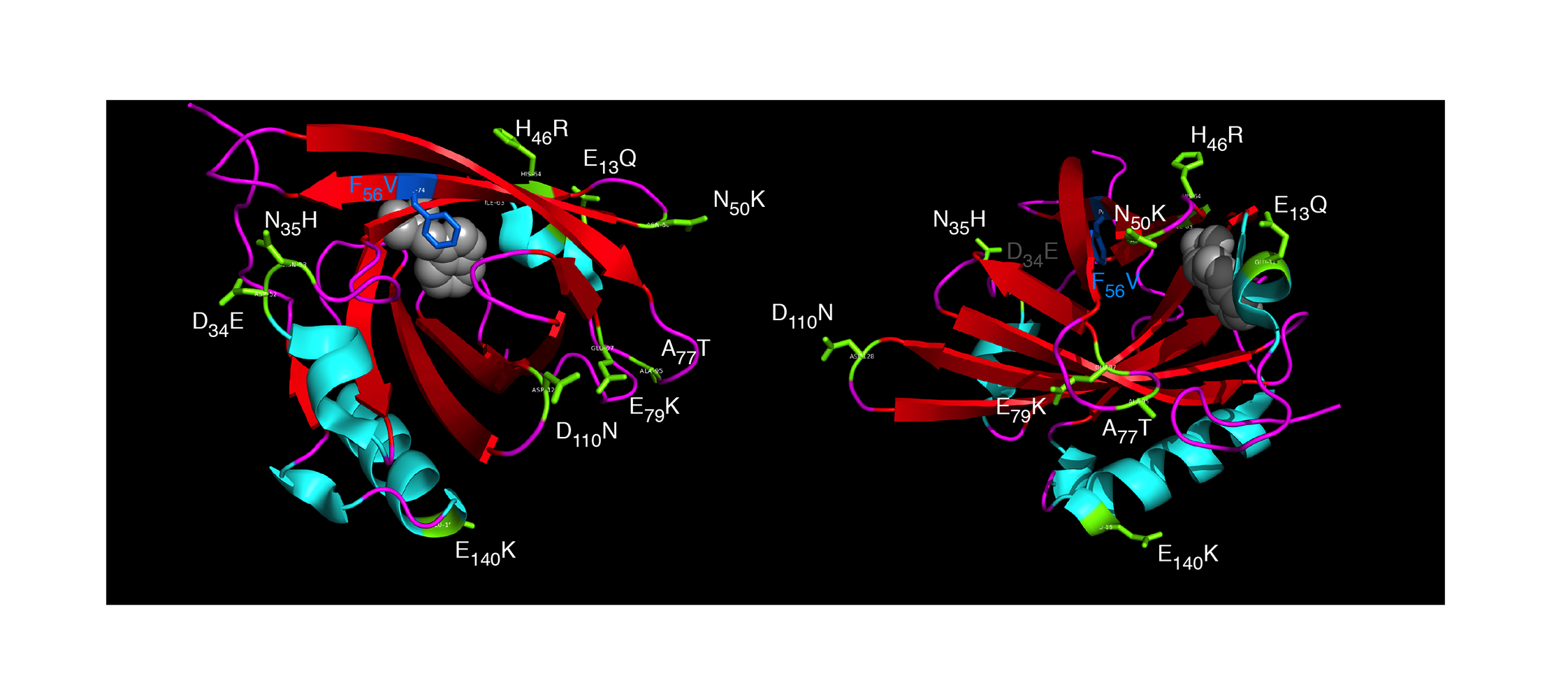

Supplement: S2 Fig — Ten of the eleven amino acid variants occur on the outside of the protein consistent with a role for the variants in differential binding between MUPs and type 2 vomeronasal receptors (V2Rs). (TIF) [file pgen.1005891.s002.tif]

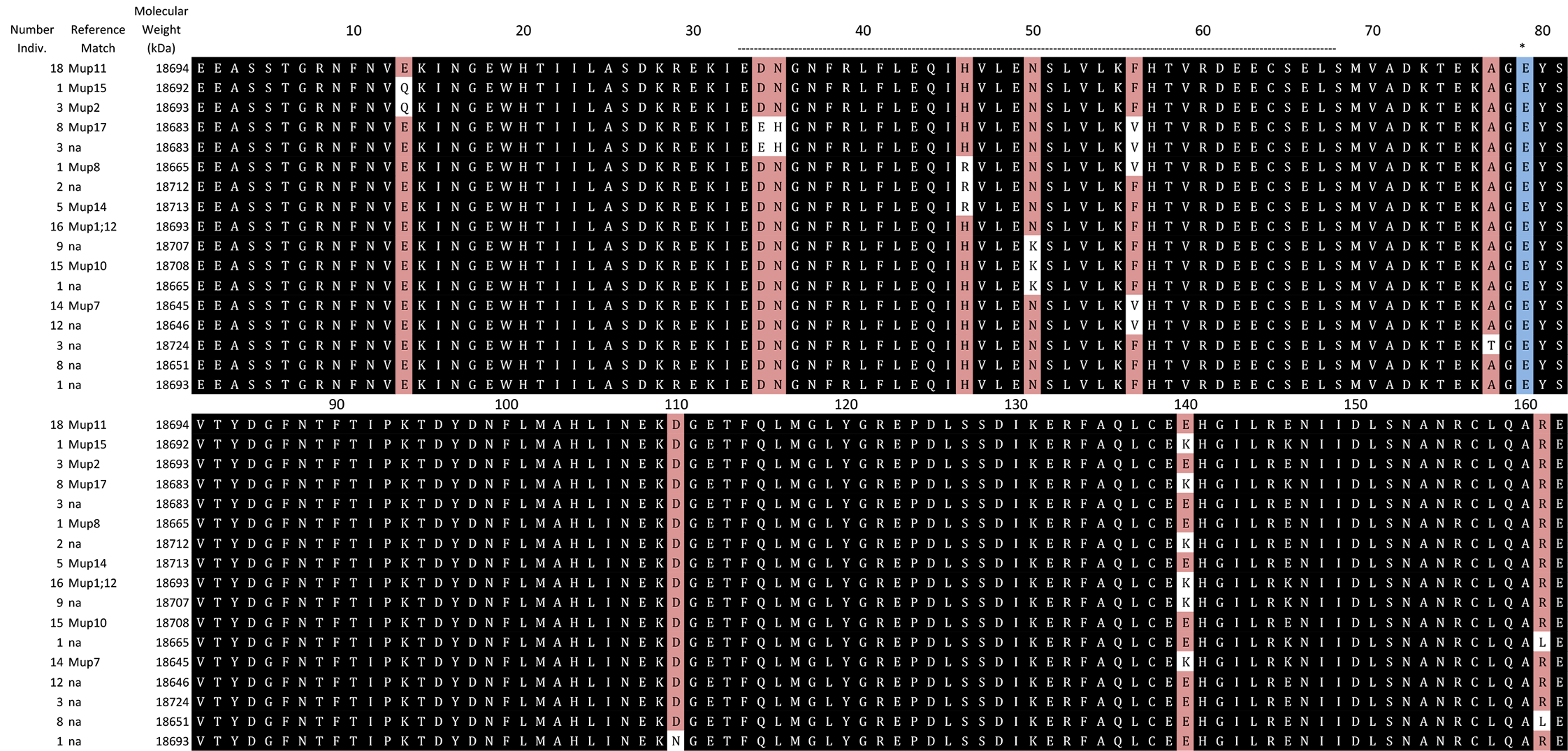

Supplement: S3 Fig — The mice in our sample collectively excrete 17 unique proteins in their urine, many of which are not found in the reference genome. The signal peptide of exon 1, the sequence of which is identical between some genes, is not shown here. The dashed line indicates the region used to estimate the expected molecular weight proportions from RNA data. The * denotes a site which is variable in the DNA data but is not variable in the RNA dataset. (TIF) [file pgen.1005891.s003.tif]

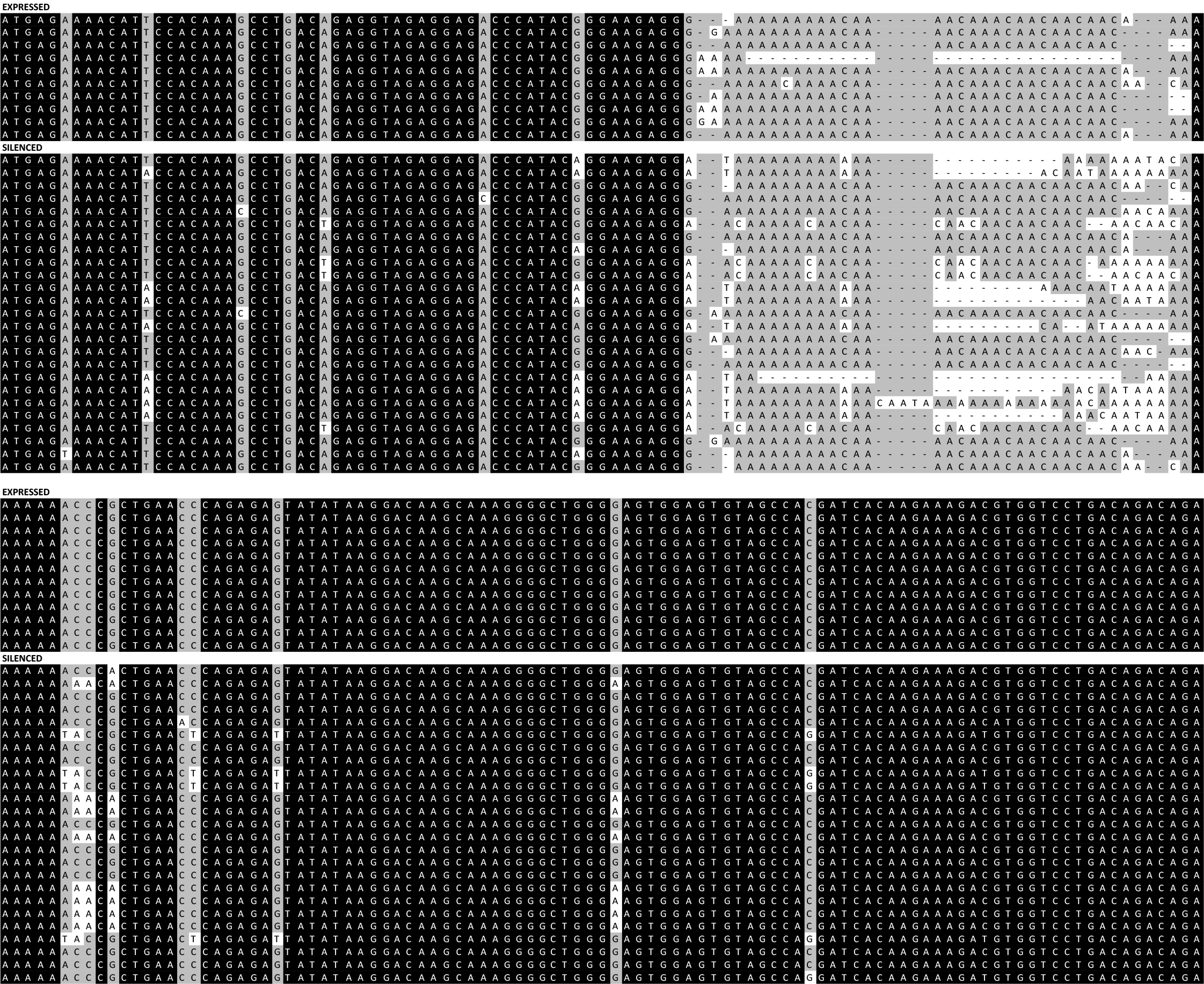

Supplement: S4 Fig — An illustrative sample of promoter variants found just upstream of the start codon in our sample. Variable sites are highlighted in grey. The expressed sequences (top group) show little variation compared to the silenced sequences (bottom group). The large, variable poly-A region lies immediately upstream of the transcription initiation site. (TIF) [file pgen.1005891.s004.tif]

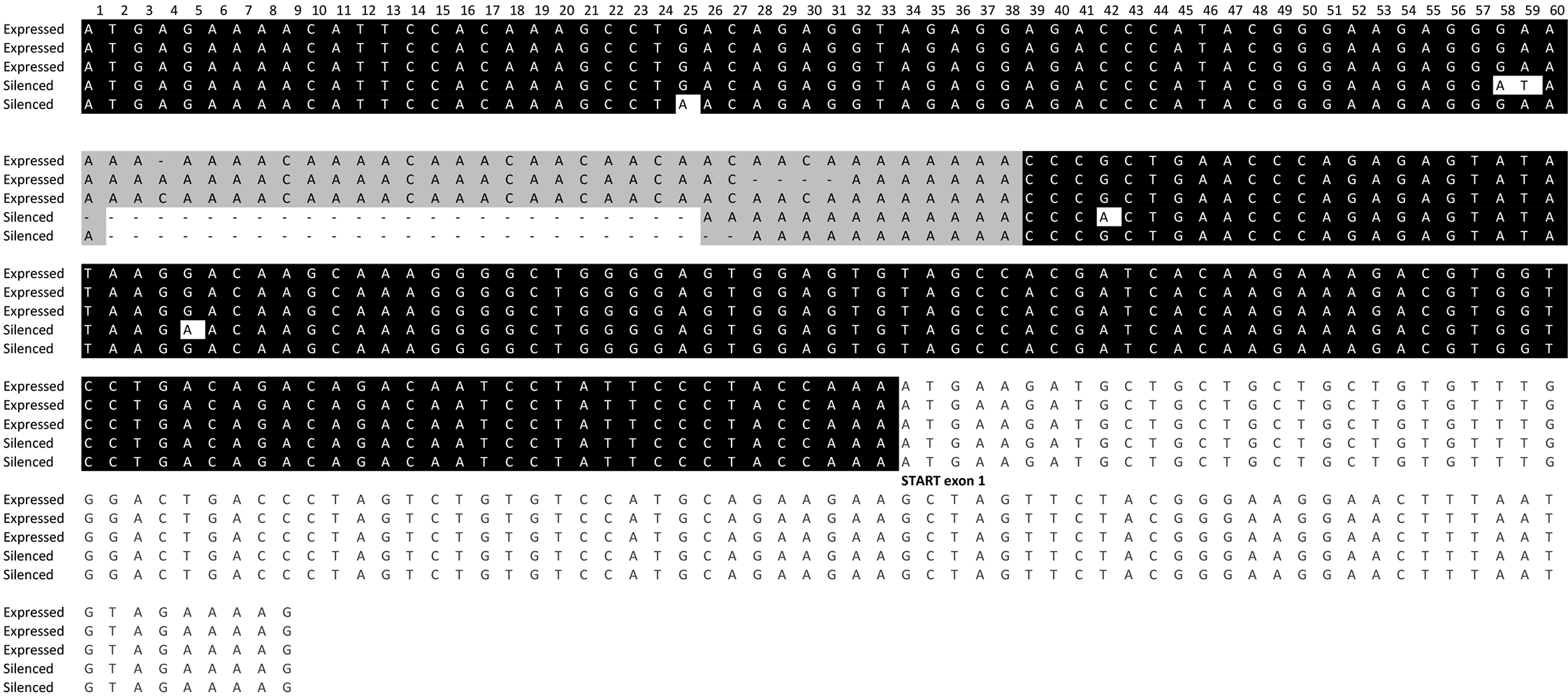

Supplement: S5 Fig — Promoter sequences associated with the same exon 1 sequences (large white block) from a single individual suggest that some copies of multi-copy genes are silenced while others are expressed. The exact expression state of the promoter shown here is not known as it is not possible to differentiate among the copies in the RNA pool. The expression state is inferred based on the similarity of the promoter sequences to sequences of known expression status. (TIF) [file pgen.1005891.s005.tif]
