# Supplementary material for: Selection on Coding and Regulatory Variation Maintains Individuality in Major Urinary Protein Scent Marks in Wild Mice
Source: PLoS Genet. 2016 Mar 3;12(3):e1005891. doi: 10.1371/journal.pgen.1005891 (PMC4777540; doi:10.1371/journal.pgen.1005891)
Supplement: S1 Table — (DOCX) [file pgen.1005891.s006.docx]

Table S1: Samples used in this study

| Sample ID | Father | Father's Location | Mother | Mother's Location |
| --- | --- | --- | --- | --- |
| TAS285 | TAS87 | 53° 26.611' N,113° 21.729' W | TAS92 | 53° 26.611' N,113° 21.729' W |
| TAS286 | TAS120 | 53° 29.014' N,114° 06.245' W | TAS125 | 53° 27.198' N,113° 57.943' W |
| TAS287 | TAS152 | 53° 23.971' N,113° 57.579' W | TAS130 | 53° 23.971' N,113° 57.579' W |
| TAS288 | TAS165 | 53° 34.818' N,114° 00.287' W | TAS111 | 53° 31.350' N,113° 37.466' W |
| TAS289 | TAS150 | 53° 24.472' N,113° 54.427' W | TAS134 | 53° 24.472' N,113° 54.427' W |
| TAS290 | TAS158 | 54°12.368' N,113° 39.136' W | TAS157 | 54°12.368' N,113° 39.136' W |
| TAS291 | TAS122 | 53° 22.060' N,113° 20.453' W | TAS123 | 53° 22.060' N,113° 20.453' W |
| TAS292 | TAS118 | 53° 29.014' N,114° 06.245' W | TAS116 | 53° 29.014' N,114° 06.245' W |
| TAS293 | TAS186 | 53° 14.921' N,113°42.566 ' W | TAS69 | 53° 30.367' N,113° 32.802' W |
| TAS294 | TAS184 | 53° 22.033' N,113° 51.408' W | TAS167 | 53° 22.033' N,113° 51.408' W |
| TAS295 | TAS101 | 53° 26.386' N,113° 04.173' W | TAS93 | 53° 26.386' N,113° 04.173' W |
| TAS296 | TAS156 | 53° 30.768' N,113° 52.365' W | TAS102 | 53° 23.738' N,113° 11.374' W |
| TAS298 | TAS151 | 53° 23.737' N,113° 54.695' W | TAS141 | 53° 23.737' N,113° 54.695' W |
| TAS338 | TAS109 | 53° 30.768' N,113° 52.365' W | TAS114 | 53° 30.768' N,113° 52.365' W |
| TAS339 | TAS154 | 53° 28.184' N,113° 53.278' W | TAS96 | 53° 51.691' N,113° 18.164' W |
| TAS340 | TAS85 | 53° 30.062' N,113° 32.082' W | TAS84 | 53° 30.062' N,113° 32.082' W |
| TAS359 | TAS127 | 53° 23.702' N,113° 56.268' W | TAS91 | 53° 24.522' N,113° 19.141' W |
| TAS360 | TAS122 | 53° 22.060' N,113° 20.453' W | TAS123 | 53° 22.060' N,113° 20.453' W |
