# Supplementary material for: Selection on Coding and Regulatory Variation Maintains Individuality in Major Urinary Protein Scent Marks in Wild Mice
Source: PLoS Genet. 2016 Mar 3;12(3):e1005891. doi: 10.1371/journal.pgen.1005891 (PMC4777540; doi:10.1371/journal.pgen.1005891)
Supplement: S2 Table — (DOCX) [file pgen.1005891.s007.docx]

Table S2: Timing of urine and RNA sampling

| Mouse ID | Born | RNA sampled (death) | Age at death | Urine1 | Urine2 | Days between urine samples | Age at Urine sampling | Days between urine and RNA sampling |
| --- | --- | --- | --- | --- | --- | --- | --- | --- |
| TAS285 | 11/20/12 | 6/17/13 | 209 | 5/29/13 | 5/10/13 | 19 | 190 | 19 |
| TAS286 | 9/24/12 | 6/17/13 | 266 | 5/13/13 | 5/29/13 | 16 | 231 | 35 |
| TAS287 | 10/1/12 | 6/17/13 | 259 | 5/29/13 | 5/29/13 | 0 | 240 | 19 |
| TAS288 | 10/1/12 | 6/18/13 | 260 | 6/9/13 | 5/10/13 | 30 | 251 | 9 |
| TAS289 | 11/18/12 | 6/18/13 | 212 | 5/29/13 | 6/5/13 | 7 | 192 | 20 |
| TAS290 | 11/5/12 | 6/18/13 | 225 | 6/5/13 | 6/5/13 | 0 | 212 | 13 |
| TAS291 | 11/10/12 | 6/18/13 | 220 | 5/29/13 | 5/13/13 | 16 | 200 | 20 |
| TAS292 | 11/11/12 | 6/18/13 | 219 | 5/29/13 | 5/3/13 | 26 | 199 | 20 |
| TAS293 | 10/30/12 | 6/18/13 | 231 | 6/9/13 | 6/3/13 | 6 | 222 | 9 |
| TAS294 | 10/2/12 | 6/18/13 | 259 | 6/5/13 | 5/15/13 | 21 | 246 | 13 |
| TAS295 | 11/18/12 | 6/18/13 | 212 | 6/9/13 | 5/10/13 | 30 | 203 | 9 |
| TAS296 | 10/1/12 | 12/27/13 | 452 | 5/29/13 | 5/10/13 | 19 | 240 | 212 |
| TAS298 | 10/1/12 | 12/27/13 | 452 | 6/5/13 | 5/29/13 | 7 | 247 | 205 |
| TAS338 | 11/20/12 | 7/17/13 | 239 | 5/29/13 | 5/29/13 | 0 | 190 | 49 |
| TAS339 | 10/1/12 | 7/17/13 | 289 | 6/9/13 | 6/3/13 | 6 | 251 | 38 |
| TAS340 | 11/18/12 | 7/17/13 | 241 | 6/3/13 | 6/9/13 | 6 | 197 | 44 |
| TAS359 | 11/18/12 | 10/8/13 | 324 | 6/9/13 | 6/3/13 | 6 | 203 | 121 |
| TAS360 | 11/10/12 | 10/8/13 | 332 | 6/9/13 | 6/3/13 | 6 | 211 | 121 |
