# Supplementary material for: Selection on Coding and Regulatory Variation Maintains Individuality in Major Urinary Protein Scent Marks in Wild Mice
Source: PLoS Genet. 2016 Mar 3;12(3):e1005891. doi: 10.1371/journal.pgen.1005891 (PMC4777540; doi:10.1371/journal.pgen.1005891)
Supplement: S3 Table — (DOCX) [file pgen.1005891.s008.docx]

Table S3: Peripheral *Mup* variants

| Chrom. | POS | Gene | ID | REF | ALT | ALT COUNT | QUAL |
| --- | --- | --- | --- | --- | --- | --- | --- |
| chr4 | 59959947 | Mup4 | Intron | T | C | 36 | 999 |
| chr4 | 60005935 | Mup6 | exon (D > V) | A | T | 11 | 999 |
| chr4 | 60005992 | Mup6 | exon (I > N) | T | A | 7 | 999 |
| chr4 | 61832868 | Mup5 | Intron | A | T | 36 | 999 |
| chr4 | 61833309 | Mup5 | Intron | A | G | 2 | 999 |
| chr4 | 61833461 | Mup5 | Intron | T | C | 2 | 999 |
| chr4 | 61833467 | Mup5 | Intron | G | A | 2 | 999 |
| chr4 | 61833476 | Mup5 | Intron | C | G | 2 | 999 |
| chr4 | 61833488 | Mup5 | Intron | C | A | 2 | 999 |
| chr4 | 61833586 | Mup5 | Intron | A | G | 2 | 999 |
| chr4 | 61833814 | Mup5 | Intron | A | C | 2 | 999 |
| chr4 | 61833841 | Mup5 | Intron | G | A | 2 | 999 |
| chr4 | 61833872 | Mup5 | Intron | C | T | 2 | 999 |
| chr4 | 61833910 | Mup5 | Intron | G | A | 2 | 999 |
| chr4 | 61833929 | Mup5 | Intron | C | T | 2 | 999 |
| chr4 | 61833934 | Mup5 | Intron | T | A | 2 | 999 |
| chr4 | 61833972 | Mup5 | Intron | A | T | 2 | 999 |
| chr4 | 61834014 | Mup5 | Intron | T | G | 2 | 999 |
| chr4 | 61834074 | Mup5 | Intron | A | G | 2 | 999 |
| chr4 | 61834138 | Mup5 | Intron | G | C | 2 | 999 |
| chr4 | 61834345 | Mup5 | Intron | A | G | 2 | 999 |
| chr4 | 61835007 | Mup5 | Intron | A | G | 2 | 999 |
| chr4 | 61835236 | Mup5 | Promoter | C | A | 2 | 147 |
| chr4 | 62052062 | Mup20 | Intron | T | C | 33 | 999 |
| chr4 | 62052283 | Mup20 | Intron | A | G | 1 | 999 |
| chr4 | 62052319 | Mup20 | Intron | A | G | 10 | 999 |
| chr4 | 62052850 | Mup20 | Intron | C | T | 1 | 105 |
| chr4 | 62053358 | Mup20 | Intron | G | A | 2 | 159 |
| chr4 | 62053368 | Mup20 | Intron | A | C | 21 | 999 |
| chr4 | 62053552 | Mup20 | exon (H > P) | A | C | 2 | 999 |
| chr4 | 62053878 | Mup20 | Intron | A | G | 27 | 999 |
