# Supplementary material for: Selection on Coding and Regulatory Variation Maintains Individuality in Major Urinary Protein Scent Marks in Wild Mice
Source: PLoS Genet. 2016 Mar 3;12(3):e1005891. doi: 10.1371/journal.pgen.1005891 (PMC4777540; doi:10.1371/journal.pgen.1005891)
Supplement: S4 Table — (DOCX) [file pgen.1005891.s009.docx]

Table S4: Primers

|  | Forward | Reverse |
| --- | --- | --- |
| MupPrimer1 | 5'-TTCTCTTGAACACCACTGTTT | 5'-GGAAGGATCTTGGTTACAGGA |
| MupPrimer2 | 5'-GATTCCAAGCCACATCCAAA | 5'-ATGCAGCAGGTGTCATGCTC |
| MupPrimer3 | 5'- AGGAACAATCCTTGGCCTC | 5'-GTTGTGGGAGGGAGGTATT |
